# Patient-reported experiences of people living with a rare disease with secondary and tertiary care: a scoping review

**DOI:** 10.1186/s13023-026-04368-5

**Published:** 2026-11-04

**Authors:** Rita Francisco, Michel Wensing, Jennifer Jones, Jessie Dubief

**Affiliations:** 1 EURORDIS – Rare Diseases Europe, Paris, France; 2 Heidelberg University, Heidelberg, Germany; 3 Department of General Practice and Health Services Research, Medical Faculty, Heidelberg University, Heidelberg, Germany; 4 Genetic Alliance UK, London, UK; 5 Department of Population Health Sciences, University of Leicester, Leicester, UK

**Keywords:** Rare diseases, Healthcare, Patient experience, Patient reported experience measures (PREMs), Secondary care, Tertiary care, Scoping literature review

## Abstract

**Background:**

People living with rare diseases (PLWRD) are a heterogenous population facing common healthcare challenges. Continued access to integrated, expert healthcare is a top medical need and policy ask for PLWRD and their carers. Patient experience is a valued healthcare quality indicator with patient-reported experience measures (PREMs) emerging as serviceable instruments. To map out the experiences of PLWRD within secondary and/or tertiary healthcare settings, we conducted a scoping literature review by searching Medline, Embase and CINAHL for papers in English and set in Western countries, published from January 2005 to September 2022.

**Results:**

A total of 61 full-text peer-reviewed articles and conference abstracts were included and mapped into a first definition of PLWRD-reported experience with secondary and tertiary care structured into 11 domains and 71 categories. Domains were ‘Access’, ‘Comprehensiveness’, ‘Continuity’, ‘Coordination’, ‘Patient-centredness’, ‘Safety’, ‘Self-management support’, ‘Trust’, ‘Overall perceived quality of care’, ‘Diagnosis & Treatment/Interventions’ and ‘Service aspects of care’. Forty-two questionnaires were reported in the included papers. None of them cover all healthcare experience domains and categories nor were they fully validated across rare diseases (RD). Most studies were set in English-speaking countries, did not disclose participant ethnicity, and focused on individual RD, collectively covering less than 2% of the known 6,000 RD. Patient-reported experiences were mainly captured for adult patients, while proxy studies predominantly targeted parents of children with RD. Adolescents living with RD were the least represented age group. To build upon these findings and agree on a healthcare experience framework of secondary and tertiary healthcare for PLWRD, the RD community must be heard. The authors make five recommendations for consulting the community, finalising a conceptual framework and validating RD-PREMs, namely to (1) *Involve multiple stakeholders*, (2) *Increase RD diversity*, (3) *Cater for all age groups*, (4) *Consider gender and ethnicity*, (5) *Ensure geographic representativeness*.

**Conclusions:**

This study provides actionable evidence and guidance to validate a healthcare experience framework for PLWRD which can inform the creation of generic RD-PREMs feeding a quality improvement system at hospital, regional, country and European levels, thus ensuring equitable access to integrated care for PLWRD.

**Supplementary Information:**

The online version contains supplementary material available at https://doi.org/10.1186/s13023-026-04368-5.

## Introduction

In Europe, rare diseases (RD) have a point prevalence below 5 cases per 10,000 inhabitants. While each disease impacts only a small number of people, over 6,000 RD have been identified to date and it is estimated that 30 million people in Europe and 300 million worldwide live with a RD [1]. RD are often complex and chronic, requiring continued expert healthcare. But expertise is scarce and scattered [2, 3] which creates common healthcare challenges for people living with a RD (PLWRD), one being access to high-quality, integrated care. Integrated care can be defined as a person-centred, multidisciplinary, and continuous approach that bridges health and social care for both PLWRD and their family carers [4]. Ensuring access to long-term, integrated, and high-quality care for PLWRD and their families are two top policy recommendations aimed at tackling RD healthcare inequalities across Europe [5].

PLWRD have higher unmet healthcare support needs and worse healthcare experiences when compared to those with common disorders like cancer, diabetes, cardiovascular and respiratory conditions [6–8]. PLWRD and family carers in Europe have flagged significant room for improvement in health and social service delivery, having reported inadequate access to follow-up consultations and to appropriate psychological and social support [9–11]. Ineffective care coordination has also been frequently experienced by PLWRD, as reported in a study in the United Kingdom (UK) [12]. In contrast, access to specialised care in centres of expertise has been linked to better healthcare experiences for PLWRD across Europe [9–11]. Among PLWRD and their relatives there is a strong perceived relationship between quality of care and quality of life [6, 7]. Hence, exploring and understanding PLWRD and their carers’ experiences with healthcare are particularly relevant. This literature review focuses on the experiences of PLWRD with expert care, in secondary or tertiary healthcare settings.

Patient experience has emerged as a key independent dimension of healthcare quality [13–15]. Continuous and consistent assessment of patient experiences with healthcare guide meaningful health decision-making, drive quality and outcome improvement, foster accountability and transparency, as well as allow comparison-making across medical centres, countries, and diseases [16, 17]. Patient reported experience measures (PREMs) are questionnaire-based instruments that assess the process of care as experienced by patients or family carers [18–20]. PREMs can be insightful monitoring and assessment tools of integrated care for PLWRD, ensuring healthcare delivery aligns with their needs and preferences. Results from the only Europe-wide survey [9–11] administering a PREM validated for chronic diseases (the short version of the Patient Assessment of Chronic Illness Care, or PACIC-S) to PLWRD showed that the reliability of the PACIC-S was high but its content validity was low [21], because it did not explore several RD-relevant aspects, including access to emotional support.

### Aims and objectives

By examining the current landscape of RD patient experience with secondary and/or tertiary care, this scoping review aims to:

- Identify and extract the domains and categories of healthcare experience included in empirical studies exploring the views of PLWRD and their family carers.
- Inform a first structured definition of healthcare experience for PLWRD, outlining whether and/or which RD-specific aspects it includes.
- Map and assess existing questionnaires against the identified healthcare experience domains and categories to determine whether they allow full and comprehensive capture of PLWRD’s healthcare experience.

## Methods

**Fig. 1:**
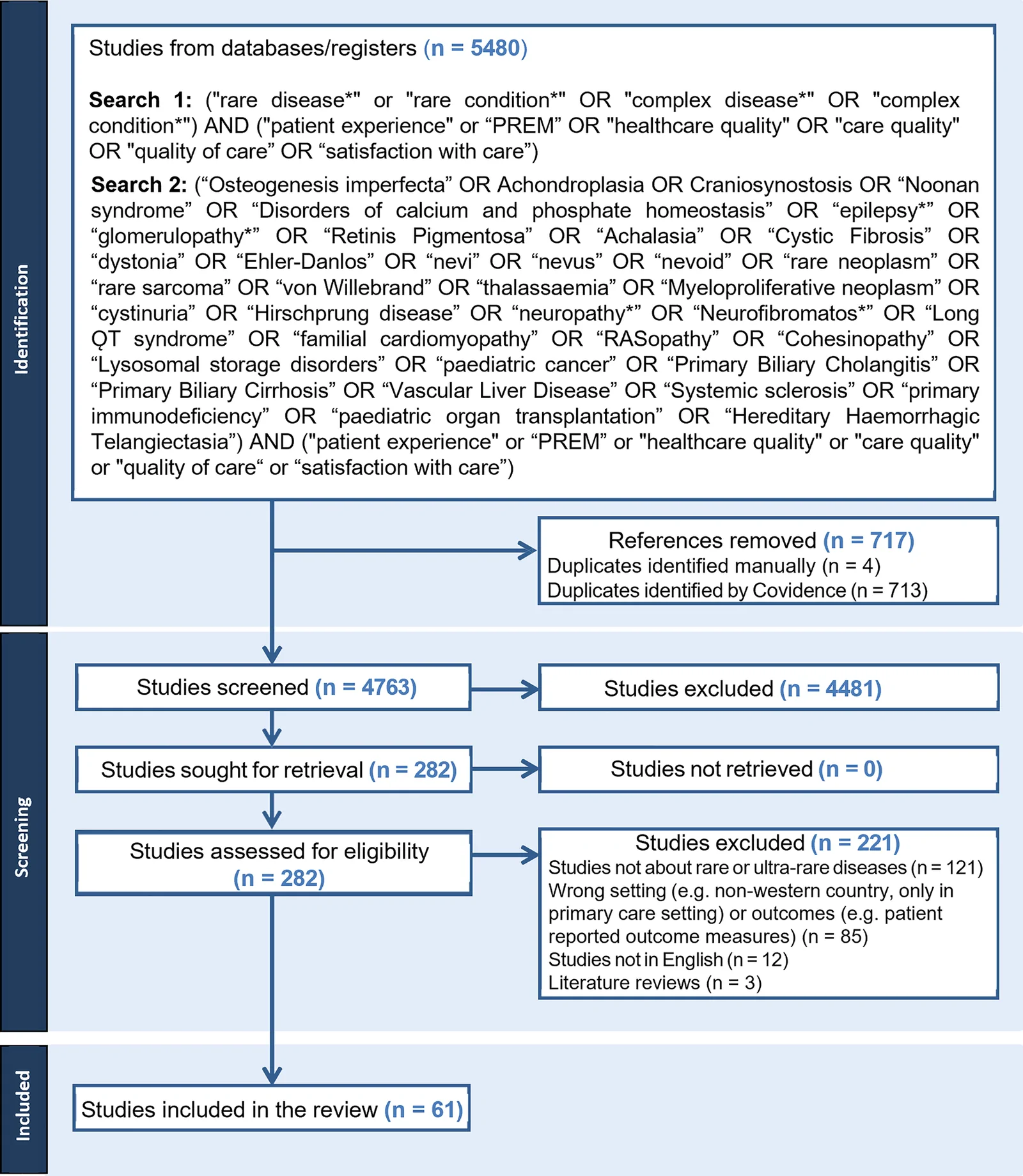
Flow diagram outlining article identification, screening and selection processes

We used a scoping review methodology, following the PRISMA Extension for Scoping Reviews [22]. Searches in Medline (through PubMed), Embase, and CINAHL were conducted between June and September 2022 using a search string that combined: (a) keywords for rare or complex diseases, and for two of the most prevalent conditions by therapeutic area as defined and covered by each of the 24 European Reference Networks (ERNs), (b) keywords for patient experience or healthcare quality (see Fig. 1 and additional file 1). Papers published between January 2005 and September 2022 were captured in the search.

Eligibility criteria were adapted from the Population, Intervention/Exposure, Comparators, Outcomes and Study characteristics (PICOS) questions [23]. Studies were included if they [1] were about RD, captured and reported the views and experiences of PLWRD and/or their family members/carers [2]; reported qualitative and/or quantitative empirical research on experiences with secondary and/or tertiary healthcare among PLWRD from a patient and/or carer perspective [3]; were set in or reported Western countries, defined as Europe and those countries settled by Europeans (the United States, Canada, Australia and New Zealand) [4]; were written in English, and [5] were published in peer-reviewed journals. Studies that (i) (only) reported on health outcomes or aspects of daily living; (ii) only assessed primary and/or remote care, or (iii) exclusively focused on the transition from child to adult care services were excluded.

Paper screening was conducted on the Covidence online platform [24] by two independent reviewers who evaluated all articles following a two-step process: (i) the title and abstract of the retrieved articles were screened; (ii) full texts of all the potentially relevant papers were checked against the eligibility criteria. Article inclusion or exclusion was regularly discussed among the research team until consensus was reached, or a third reviewer resolved disagreements.

Data extraction was performed on Covidence by two blinded reviewers. Consensus was reached and validated by a third reviewer and/or through group discussions, when needed. The data extraction template was structured into 3 main sections (Additional file 2): (i) general information of the paper (title, year of publication, authors, target population, study type, disease/disease group); (ii) methods used: quantitative, qualitative, quantitative-qualitative (questionnaire-based studies containing and reporting closed and open-ended questions) and mixed-methods (applying two consecutive data collection methods); (iii) 11 domains and 71 categories of healthcare experience. Domains 1 to 9 (‘Access’, ‘Comprehensiveness’, ‘Continuity’, ‘Coordination’, ‘Patient-centredness’, ‘Safety’, ‘Self-management support’, ‘Trust’, ‘Overall perceived quality of care’) were informed by the PREM section of the Patient-Reported Indicator Surveys (PaRIS) study [25, 26]. The scope of each domain was defined based on the research team’s expertise and domain categories were assigned to systematise and provide more granularity to data extraction. We added domains 10 and 11 (‘Diagnosis & Treatment/Interventions’ and ‘Service aspects of care’) as well as 29 categories (to the initial 42, bringing the number of categories to 71) during data extraction to improve capture and reporting of the evidence found in the literature. These additional domains and categories emerged through a gradual, inductive process resulting from the iterative extraction and analysis of the data reported in the peer-reviewed publications included in this review. Their addition was discussed and validated by all co-authors based on their knowledge of RD and theoretical frameworks of healthcare experience. The extracted data was exported to Excel where data filters and sum formulas were applied, and percentages calculated for description.

## Results

The search identified 4,763 articles, of which 282 underwent full-text screening: 61 articles met the inclusion criteria (46 full-text papers and 15 conference abstracts). The PRISMA diagram illustrating this process is presented in Fig. 1. Papers derived from the same study but reporting different results included (i) four articles reporting the development and administration of a cystic fibrosis (CF)-specific PREM [27–30], (ii) three papers outlining the design, implementation and results of the administration of a systemic sclerosis (SS) online questionnaire to measure quality of care and determine care quality indicators from a patient perspective [31–33], and (iii) three articles produced under the banner of the European DISABKIDS project describing the creation, adaptation and results of two validated PREMs, the Child Healthcare measure-satisfaction, utilization, and needs (CHC-SUN) proxy-instrument and the Youth Healthcare measure-satisfaction, utilization, and needs (YHC-SUN) questionnaire, a self-reported version for adolescents [34–36]. Additional file 3 details the main characteristics of the included papers, including authors, year of publication, country, disease(s), number of participants and study type(s), among other.

### Paper characterisation: study design, location, clinical focus and study populations

Of the 61 included papers, 61% (37/61) exclusively reported quantitative methods and findings, while 21% (13/61) only reported qualitative methods. Mixed methods and qualitative-quantitative tools respectively account for 10% (6/61) and 8% (5/61) of the papers (Additional file 3). Quantitative methods were limited to questionnaires (48 papers [27–35, 37–75]): while qualitative methods included interviews (14 papers [31, 32, 42, 43, 76–85]), focus groups (8 papers [27, 30–32, 35, 80, 86, 87]), narrative/open-ended questions (5 papers [37–41]), and observations (one paper [76]): and a working conference [32]. Validation activities were reported in 67% (41/61) of the papers, distributed as follows: 22 quantitative studies, 10 qualitative studies, 5 mixed-methods studies, and 4 qualitative-quantitative studies. Table 1 outlines the specific validation measures described in these articles. PLWRD, family carers and/or patient advocates and organisations were involved in 49% of the papers (30/61) at varying levels and capacities in study/instrument design, participant recruitment and/or data management (Table 1).

**Table 1:** Validation (*n* = 41 papers) and patient involvement (*n* = 30 papers) measures

Type of validation and patient involvement measure applied in studies reported by papers	Number of papers	Reference (No)
**Use and/or adaptation of existing validated instruments**		
Generic instruments	14	[28, 29, 31, 33, 36, 43–45, 52, 53, 58, 64, 70, 75]
Disease-specific instruments	1	[62]
**Conducted literature reviews and/or used patient experience healthcare frameworks**^**a**^	9	[34, 35, 50, 51, 58, 63, 77, 80, 85]
**Internal consistency & other statistical tests**^**b**^	11	[30, 34, 44, 48, 51, 52, 56, 59, 62, 64, 73]
**Multi-phased analysis**^**c**^	11	[37, 39, 41, 43, 76, 77, 80, 83, 84, 87, 88]
**Several researchers involved in analysis and cross-checking analysis with interviewees**^**c**^	11	[32, 39, 43, 77, 78, 80, 83–85, 87, 88]
**Patients and carers involvement in the research process**^**d**^	**30**	
Patient participation (contribute to participant recruitment)	8	[44, 50, 54, 55, 60, 75, 78, 83]
Patient engagement (review documents/instrument content, be involved in pilot testing of instruments and guides and/or in priority-setting exercises)	23	[27–30, 32–34, 36, 38, 42, 44, 46, 48, 50, 51, 56, 58, 60, 70, 77, 80, 84, 88]
Patient involvement/partnership (being full members of advisory groups and/or partners by co-designing questions and data handling)	7	[32, 33, 54, 56, 59, 63, 75]

Legend: ^a^ – Encompasses all study types; ^b^ – Only applied to papers with a quantitative component; ^c^ – Only applicable to studies with a qualitative component; ^d^ - Patient involvement levels established based on [4] and adapted to the context of this scoping review [89]

Twenty-six of the 61 included papers describe patient-reported experiences. Eighteen report the experience with secondary and tertiary healthcare of adult patients only (defined as aged over 18 years-old), 7 report the experience of adults and adolescents, and one paper includes patients of all ages. The experiences of children living with a RD were captured by proxy of their parents in 11 papers. The remaining 24 papers concerned both patients and family carers: 14 included adult patients and parents of children living with a RD, four included children and/or adolescents and their parents, and 6 included patients of all ages and their family carers, with the latter encompassing parents and other relatives (Table 2). Overall, the least represented age group are adolescents who were only included in 15 papers.

**Table 2:** Patient and proxy-reported experiences (*n* = 61 papers)

Studies Age groups	Number of papers	Reference (No)
**Only patient-reported experiences**	**26**	
Adult patients	18	[31–33, 40, 47–49, 64, 65, 67, 68, 75, 80, 81, 83–86]
Adult and adolescent patients	7	[36, 41, 42, 51, 52, 54, 69]
All ages	1	[88]
**Only proxy-reported experiences**	**11**	
Parents of children living with a rare disease	11	[34, 35, 37, 43, 45, 53, 60, 61, 66, 72, 77]
**Both patient and proxy-reported experiences**	**24**	
Adult patients and parents of children living with a rare disease	14	[27, 30, 39, 44, 46, 50, 55, 58, 59, 62, 63, 71, 76, 82]
Child and/or adolescent patients and their parents	4	[56, 57, 73, 87]
All ages (patients and carers)	6	[28, 29, 38, 70, 74, 78]

In terms of geographical coverage, single-country studies represent 85% (52/61) of all included papers. The most represented countries are the UK (*n* = 19), the USA (*n* = 12), Germany (*n* = 12) and the Netherlands (*n* = 10) (Table 3).

**Table 3:** Countries where studies were set (*n* = 61* papers)

Country where the study was undertaken	Number of papers	Reference (No)
**Europe**	**44**	
UK	19	[34, 35, 38, 40, 41, 46, 50, 52, 54, 55, 57, 64, 66, 67, 69, 71, 75, 76, 78]
Germany	12	[27–30, 34–36, 47, 51, 52, 55, 75]
Netherlands	10	[31–35, 52, 54, 55, 72, 75]
France	7	[34, 49, 52, 54, 55, 63, 75]
Italy	6	[39, 54, 55, 59, 74, 75]
Sweden	4	[34, 35, 48, 52]
Greece	4	[34, 35, 53, 75]
Spain	3	[44, 54, 88]
**Outside Europe**	**18**	
US	12	[45, 56, 58, 60–62, 65, 70, 73, 75, 82, 87]
Australia	6	[37, 43, 68, 75, 83, 87]
Canada	4	[75, 84, 85, 87]

Paper 75 is set in different countries both inside and outside of Europe

Single disease papers account for 59% (35/61) of the selected articles while the remaining 41% (26/61) focus on multiple diseases (Table 4). The most represented diseases are CF (*n* = 23), SS (*n* = 6), inflammatory bowel disease (*n* = 5), juvenile arthritis (*n* = 4) and haemophilia (*n* = 2) (Table 4).

**Table 4:** Individual and multiple rare diseases covered by studies reported in papers (*n* = 61 papers)

Rare disease	Number of papers	Reference (No)
**Single disease**	**35**	
Cystic fibrosis (CF)*	18	[27–30, 41, 42, 53, 62, 63, 65, 68, 69, 71–74, 76, 82]
Systemic sclerosis (SS)*	3	[31–33]
Inflammatory bowel disease	2	[45, 51]
Epithelioid haemangioendothelioma	1	[75]
Primary Biliary Cholangitis*	1	[67]
Craniosynostosis*	1	[61]
Poland syndrome	1	[59]
Alkaptonuria	1	[54]
Paediatric hereditary angioedema	1	[57]
Pseudoxanthoma elasticum	1	[49]
Pulmonary Arterial Hypertension	1	[48]
Primary lymphoedema	1	[47]
Multiple myeloma	1	[81]
Alström syndrome	1	[38]
Myelofibrosis*	1	[39]
**Multi-disease**	**26**	
Rare cancers	5	[55, 60, 64, 77, 84]
Rare neurological diseases	2	[43, 85]
Rare blood diseases	2	[58, 70]
Rare metabolic diseases	1	[88]
Rare skin diseases	1	[80]
Rare heart diseases	1	[83]
Rare kidney diseases	1	[87]
Rare and complex connective tissue and musculoskeletal diseases	1	[86]
Rare endocrine diseases	1	[52]
Rare lung diseases	1	[66]
Other (several)	10	[34–37, 40, 44, 46, 50, 56, 78]

* Specific keywords for these diseases were included in the literature search string used to query the databases (Additional file 1)

Participants’ ethnicity and/or race was only recorded and/or reported in 9 papers, 8 of them being set in the US and Canada [45, 56, 60–62, 65, 70, 85], and one in the UK [64].

### People living with a rare disease (PLWRD) experience with secondary and tertiary healthcare

Of the 11 healthcare experience domains for PLWRD defined here, the three most often explored in the included papers are ‘Self-management’ (89%, 54/61), ‘People-centredness’ (87%, 53/61), and ‘Comprehensiveness’ (80%, 50/61). Then come ‘Coordination’ (72%, 44/61), ‘Access’ and ‘Overall perceived quality of care’ (69%,42/61 each), ‘Continuity’ (57%,35/61) and ‘Diagnosis & Treatment/Interventions’ (54%,33/61). The least explored domains are ‘Service aspects of care’ (33%, 20/61), ‘Trust’ (23%, 14/61) and ‘Safety’ (13%, 8/61); 75% (6/8) of the papers that broach the ‘Safety’ domain are about CF [28, 30, 56, 63, 68, 71] (Table 5).

The number of domains explored per paper varied widely from one paper which focused only on ‘People-centredness’ [72] to three articles covering all 11 domains [28, 30, 80]. Domains ‘Diagnosis & Treatment/Interventions’, ‘Coordination’, ‘Trust’ and ‘Service aspects of care’ were more frequently referenced in qualitative papers (Table 5).

Among the 71 categories of healthcare experience for PLWRD assigned to the 11 domains, the three most mentioned fall under the scope of domains ‘People-centredness’ and ‘Comprehensiveness’, namely ‘healthcare professional (HCP) communication/listening skills’ (64%, 39/61), followed by ‘access to multidisciplinary care’ (62%, 38/61) and ‘patient/carer participation/involvement in care decision-making’ (61%, 37/61). Many of the categories of the ‘Comprehensiveness’ and ‘Coordination’ domains were either restricted to or more present in qualitative papers (Table 5).

**Table 5:** Framework of rare disease patient experience in secondary and tertiary healthcare settings

Domains Categories	Number of papers	References (No) per type by methodology used				
		Quantitative (*n* = 37 papers)	Qualitative (*n* = 13 papers)	Mixed methods (*n* = 6 papers)		Qualitative-quantitative (*n* = 5 papers)
**Domains [**1**] to [**9**] have been defined based on the Patient-Reported Indicator Surveys (PaRIS) study promoted and led by OECD [**26**]**.						
**1.Access** *Ease with which patients can obtain healthcare services. It includes timely appointments, convenient location, appropriate duration, financial and physical barriers to access (e.g. for people in wheelchairs) or waiting times to receive healthcare.*	**42**	[28, 29, 33, 34, 36, 44, 47, 48, 50–53, 55, 56, 58–64, 68, 70, 71, 74]	[35, 76, 78, 80, 81, 83, 84, 88]		[30–32, 43]	[37–41]
1.1. Duration of appointments (little time/enough time)	20	[28, 29, 33, 36, 47, 51, 55, 56, 58, 62, 64, 70]	[76, 80, 81]		[30, 43]	[38, 39, 41]
1.2. Waiting time (in the waiting room, to get an appointment after doing tests, outside of routine visits, unexpected cancellations)	17	[28, 29, 36, 48, 50, 51, 61, 62, 64, 68, 70]	[80, 84, 88]		[30]	[37, 40]
1.3. Timing of appointments – suitability	17	[28, 29, 48, 51, 58, 62, 68]	[35, 76, 78, 83, 88]		[30]	[37, 38, 40, 41]
1.4. Location of appointments – convenience	12	[33, 44, 50, 53, 56, 60, 71]	[31, 32, 78]		*NA*	[39, 41]
1.5. Mode of appointment (in person, phone, video call) – acceptability	10	[34, 36, 52, 53, 58, 62, 74]	[81, 83]		*NA*	[37]
1.6. Insurance coverage and financial burden (parking, travelling, testing costs, access other types of financial support and reimbursement of care)	10	[34, 51, 58, 59, 61, 70]	[35, 78, 84]		*NA*	[39]
1.7. How the appointment was made (in person, by phone, online …) – easiness	4	[44, 63, 74]	[78]		*NA*	*NA*
**2.Comprehensiveness** *Coverage of all health issues in care receiving, in accordance with the biopsychosocial model of care. It includes availability of various medical specialties, monitoring tools, social, psychological and educational services, impact of the condition on the family.*	**50**	[28, 29, 33, 34, 36, 44–53, 55–63, 66–68, 70, 71, 75]	[35, 76–78, 80–88]		[30–32, 42, 43]	[37, 38]
2.1. Access to multidisciplinary care	38	[28, 29, 33, 34, 36, 45, 46, 49–53, 58–63, 66–68, 70, 71, 75]	[35, 76–78, 81, 83, 84, 86, 88]		[30–32, 43]	[38]
2.2. Psychological/emotional support (patient and/or carer)	28	[29, 34, 36, 45, 46, 50–53, 58, 60–62, 70, 75]	[35, 77, 78, 80–83, 85, 86, 88]		[30, 43]	[38]
2.3. Access to specialised providers	17	[33, 34, 36, 44, 47, 51, 55, 57, 59, 61]	[35, 83, 84, 88]		[32, 43]	[38]
2.4. Peer-support (patient organisation or other): existing peer-support, access to peer-support	16	[34, 36, 46, 47, 51, 52, 58, 59, 71]	[77, 83–85]		[32, 43]	[38]
2.5. Involvement of/access to social worker	14	[29, 34, 36, 50, 52, 53, 62, 70]	[35, 77, 78, 85, 88]		[43]	*NA*
2.6. Guidance about social services and education resources	9	[34, 36, 44, 45, 58–60]	*NA*		[43]	[37]
2.7. Guidance on procedures for disability status and fiscal benefits	7	[34, 50]	[35, 77, 78, 85]		[43]	*NA*
2.8. Access to/existence of clinical care guidelines and necessary/optimal testing/monitoring	7	[33, 63]	[82, 83, 85, 86]		[32]	*NA*
2.9. Liaison and collaboration with schools and school-related services (awareness, liaising with school nurses, learning interventions, systematic instructions in school by HCPs)	6	[34, 36, 45, 57, 61]	[35]		*NA*	*NA*
2.10. HCPs asked about the impact of the disease on patient’s/carer’s life	6	[44, 48, 51, 56, 75]	*NA*		[42]	*NA*
2.11. Supply/access to medical/physical aid equipment, home care	5	[34, 36, 53, 60]	[78]		*NA*	*NA*
2.12. Respite care and bereavement support (for family carers)	4	[34, 36, 50, 60]	*NA*		*NA*	*NA*
2.13. Care adapted to the evolving patient needs (related to age, independent living, starting a relationship, starting a job)	4	[51]	*NA*		[30, 42]	[40]
2.14. Questions on the patient’s health habits	3	[44, 56]	*NA*		[42]	*NA*
**3. Continuity** *Degree to which a series of discrete healthcare events is experienced by people as coherent and interconnected over time and consistent with their health needs and preferences (WHO, 2018). It includes having a key person to contact, easy access to information on patient’s health and rare condition, follow-up path after visits, or adaptation of healthcare and transition depending on patients’ age and stages in life.*	**35**	[28, 29, 33, 34, 36, 44–46, 48, 49, 51, 52, 56, 57, 59, 61, 66, 68, 71, 74, 75]	[35, 77, 78, 80, 81, 83–85, 88]		[30, 43]	[37, 38, 40]
3.1. Is there a named HCP who they can contact/how contactable they are	23	[28, 29, 44, 46, 48, 51, 56, 57, 59, 61, 66, 68, 71, 74, 75]	[77, 78, 80, 81, 88]		[30, 43]	[37]
3.2. Follow-up path planned after the visit/hospital discharge (including booking of regular examinations … )	13	[28, 29, 33, 44, 49, 74]	[77, 78, 84, 85, 88]		[30]	[37]
3.3. Care continuity	10	[35, 77, 78, 83–85]	*NA*		[30, 43]	[38, 40]
3.4. Access to and satisfaction with emergency care	10	[28, 34, 44, 45, 51, 57, 66]	[35, 78]		[30]	*NA*
3.5. Access to and satisfaction with transition of paediatric to adult care	5	[45, 49, 52]	[88]		*NA*	[40]
3.6. Have an alert card about their condition which can be used in emergency situations/when they are in contact with a new HCP	2	[57, 66]	*NA*		*NA*	*NA*
**4. Coordination** *Efficient collaboration and integration of different participants (including healthcare professionals and the patient) and services involved in patients’ integrated care to ensure the appropriate delivery of healthcare services. It includes collaboration and transfer of information between all professionals involved in integrated care.*	**44**	[28, 29, 33, 34, 36, 44–53, 59–61, 63, 68, 70, 71, 73, 74]	[35, 76–78, 80, 82–86, 88]		[27, 30–32, 42, 43]	[37, 38]
4.1. Team working of the HCPs involved in care (including case conferences)	24	[28, 29, 33, 44, 46, 47, 49, 51, 52, 59, 61, 63, 71]	[35, 78, 80, 82, 88]		[27, 30–32, 43]	[38]
4.2. Effectiveness of how care is coordinated	19	[34, 36, 44–46, 48, 50, 53, 63, 70]	[76, 78, 80, 82, 85, 88]		[32, 43]	[37]
4.3. Given a care plan (written/digital)	16	[33, 44, 45, 60, 71, 73, 74]	[77, 78, 82–85]		[42]	[37, 38]
4.4. Transfer of information to the local doctor: specialist/primary care centre/general practitioner (between hospitals/clinics)	13	[33, 44, 49, 51, 70, 71]	[31, 32, 78, 83, 84]		*NA*	[37, 38]
4.5. Existence of a case manager/care coordinator	10	[45, 52]	[35, 76, 78, 85, 88]		[32, 43]	[37]
4.6. Access to medical records/history/registries	8	[44, 45, 52, 63]	[78, 86]		[32]	[37]
4.7. All specialists needed to address the case were present during consultations	4	[63, 68]	*NA*		[32]	[38]
**5. People-centredness** *People’s needs, values and preferences play a central role in the delivery and organisation of healthcare. It includes clear and adapted communication, empathy, respect and partnership.*	**53**	[28, 29, 31, 34, 36, 44, 46–48, 50–52, 55, 56, 58–64, 66–75]	[35, 41, 77, 78, 80–83, 85, 87, 88]		[30–32, 42, 43]	[37–41]
5.1. HCP communication/listening skills	39	[28, 29, 33, 36, 44, 48, 50, 51, 55, 56, 59–62, 66, 68–71, 73–75]	[35, 76, 77, 80–83, 85, 87]		[30, 42, 43]	[37–41]
5.2. Patient/carer participation/involvement in care decision-making	37	[28, 33, 34, 36, 46–48, 51–53, 55, 56, 60, 62, 63, 67, 69, 70, 72, 75]	[35, 76, 77, 80–82, 85, 87, 88]		[30–32, 42]	[37, 38, 40, 41]
5.3. Preferences of patient taken into account/involved in decision-making	27	[33, 34, 36, 44, 51, 52, 56, 60, 63, 64, 67, 72, 75]	[78, 80, 81, 83, 85, 87, 88]		[30–32, 42]	[37, 38, 40]
5.4. HCP respect, empathy and friendliness	25	[29, 33, 36, 44, 50–53, 56, 58, 62, 68–70, 73]	[35, 77, 80, 81, 85, 87]		[30]	[37, 39, 41]
**6. Safety** *Prevention of medical errors and adverse events. It includes patient safety protocols, infection control measures or the establishment of a culture of safety within the healthcare facility.*	**8**	[28, 56, 63, 68, 71]	[77, 80]		[30]	*NA*
6.1. Safety of care and of care spaces (including patient segregation if/when necessary)	6	[28, 63, 68]	[77, 80]		[30]	*NA*
6.2. Infection prevention measures, e.g., hand hygiene	2	[56, 71]	*NA*		*NA*	*NA*
**7. Self-management support** *Patients are empowered to take an active role in managing their health and condition. It includes education, resources and support to enhance patients’ self-efficacy and self-care abilities.*	**54**	[28, 29, 33, 34, 36, 44–54, 56–67, 70, 73–75]	[35, 76–78, 80, 81, 83–85, 87, 88]		[27, 30–32, 42, 43]	[37–41]
7.1. Information provided on the rare condition (before/during/after diagnosis)	24	[28, 36, 44, 46–50, 52–54, 60, 61, 65, 67]	[35, 80, 88]		[30, 42, 43]	[38–40]
7.2. Information on treatment (clear info on objectives, results … )	24	[28, 29, 36, 44, 47–49, 53, 57, 61, 62, 67, 73–75]	[35, 76, 80, 87, 88]		[27, 30, 42, 43]	*NA*
7.3. Quality of the information provided (accessibility, effectiveness/accuracy, timeliness, understandability, format)	18	[33, 46, 49–51, 56–58, 60, 70]	[77, 80, 81, 88]		[32, 42]	[37, 40]
7.4. Peer-support (patient organisation or other): informed of availability, given contact	12	[33, 34, 36, 44, 46, 50, 51, 59]	[78, 84]		[43]	[38]
7.5. Information on where to turn with questions (person/website/support group)	11	[33, 44, 66]	[35, 83, 84, 88]		[32, 42, 43]	[38]
7.6. Information on tests and test results	10	[28, 29, 44, 48, 49, 51, 56, 75]	[76]		[30]	*NA*
7.7. Education/self-management programmes	8	[34, 36, 42, 47, 63]	[84]		[32]	[38]
7.8. Information on everyday management and daily life (sex life, sports)	7	[47–49, 73, 75]	*NA*		[30, 42]	*NA*
7.9. Information on elective admissions and upon hospital discharge	6	[45, 64, 66]	[85]		[30, 43]	*NA*
7.10. Information on research and registries	5	[28, 29, 63]	*NA*		[30]	[38]
7.11. Information on local therapy, rehabilitation, physiotherapy facilities	3	*NA*	[83, 87]		[43]	*NA*
7.12. Information for carers and family members	3	*NA*	*NA*		[30, 42, 43]	*NA*
7.13. Information on reimbursement of care	1	[59]	*NA*		*NA*	NA
**8. Trust** *Patients and healthcare professionals build and maintain trustful relationships. It is bidirectional and can include effective communication, transparency, and the delivery of and adherence to high-quality healthcare.*	**14**	[28, 29, 55, 56, 76]	[35, 77, 80, 83, 88]		[30]	[37–39]
8.1. Trust/confidence in the care team	14	[28, 29, 55, 56, 76]	[35, 77, 80, 83, 88]		[30]	[37–39]
**9. Overall perceived quality of care** *Overall perception of and satisfaction with the healthcare experience, including clinical excellence, inter-personal relationships and service delivery & access.*	**42**	[28, 29, 33, 34, 36, 46–56, 58, 59, 62, 63, 65, 66, 68–70, 73]	[35, 77, 80, 81, 83, 84, 86, 88]		[30–32, 42, 43]	[37–39]
9.1. Technical/Instrumental quality of care: perceived skills of the care team (including knowledge levels/expertise of the HCPs providing care)	30	[28, 29, 33, 36, 46, 49–51, 54–56, 58, 62, 63, 66, 68]	[77, 80, 81, 83, 84, 88]		[30–32, 42, 43]	[37–39]
9.2. Overall satisfaction with care	15	[29, 34, 36, 46, 47, 52, 53, 55, 58, 62, 65, 70, 73]	[35]		*NA*	[37]
9.3. Overall experience with the care received	8	[36, 47, 48, 62, 65, 69, 73]	*NA*		*NA*	[37]
9.4. Patient participation in research	7	[46, 52, 59]	[86, 88]		[43]	[38]
Domains [10] and [11] were added by the authors during data extraction.						
**10. Diagnosis and treatment/interventions** *Efficient and adapted care journey and care pathway, considering the rarity of the condition. It includes access to appropriate diagnostic tests, announcement of diagnosis, available treatment or intervention, access to available treatment or interventions.*	**33**	[28, 33, 34, 36, 44, 46, 47, 50–52, 54, 57, 59, 61, 62, 65, 67, 73, 74]	[35, 77, 78, 80, 81, 83, 84, 86–88]		[30, 43]	[38, 39]
10.1. Diagnosis (timely, misdiagnosis, length of diagnosis journey, emotional impact, feeling dismissed)	17	[33, 36, 46, 50, 54, 57, 59, 65]	[35, 78, 81, 83, 84, 86, 88]		[43]	[39]
10.2. Access to treatment (medication refills, emergency medication, inpatient treatment, home treatment, local hospital, bureaucracy)	15	[28, 34, 36, 47, 50, 51, 57, 62, 74]	[35, 78, 84, 87, 88]		[30]	*NA*
10.3. Treatment effectiveness and satisfaction (existence and/or access to guidelines or other validated tools to assess and monitor response)	13	[33, 36, 50, 52, 59, 61, 62, 67]	[80, 86, 88]		[43]	[38]
10.4. Access to appropriate diagnostic testing (e.g., genetic testing)	5	[47, 52, 55]	[84, 88]		*NA*	*NA*
10.5. Adherence to treatment (e.g., discussing/addressing barriers to adherence)	4	[34, 36, 44, 73]	*NA*		*NA*	*NA*
10.6. Treatment safety (e.g., long-time/severe side effects and/or adverse reactions)	3	[61]	[81, 84]		*NA*	*NA*
10.7. Discomfort in pre-diagnosis phase of care	2	*NA*	[88]		*NA*	[39]
**11. Service aspects of care** *Focuses on the non-clinical elements that contribute to a positive patient experience. It includes comfortable/clean facilities, non-medical staff friendliness or timely responses to inquiries.*	**20**	[28, 29, 36, 49, 51, 53, 56, 58, 59, 68, 71]	[35, 77, 80, 81, 83, 88]		[27, 30]	[39]
11.1. Premises/Facilities (cleanliness, toilets, seating, room design and furnishing, functionality of care spaces, play areas, availability of food/snacks)	16	[28, 29, 36, 51, 53, 56, 58, 68, 71]	[35, 77, 80, 83]		[27, 30]	[39]
11.2. Friendliness of support staff	6	[29, 51, 56]	[81, 88]		[30]	*NA*
11.3. Toys provided	3	[28, 29]	*NA*		[30]	*NA*
11.4. Administrative aspects of care	3	[59]	[77, 80]		*NA*	*NA*
11.5. Received reminders for appointments	2	[48, 71]	*NA*		*NA*	*NA*
11.6. Accommodation for close family	2	*NA*	[77, 83]		*NA*	*NA*

Legend: HCP – Healthcare professional; NA – Not applicable

Domains 1 to 9 come from the Patient-Reported Indicator Surveys PaRIS) framework (25,26); domain definition and list of categories were developed by the authors of this article; additional domains (‘Diagnosis & Treatment/Intervention, and ‘Service aspects of care’) were developed by the authors of this article based on the results of the included papers

### Questionnaires on people living with a rare disease’s (PLWRD) experience with secondary and tertiary healthcare

A total of 42 different questionnaires were reported to have been developed, adapted, or administered in the papers considered in this review: they are listed in Table 6 and in additional file 4, numbered from Q1 to Q42. Among them, 71% (30/42) are unvalidated, author-built questionnaires designed by adapting and/or merging validated questionnaires with author-designed questions (Q8-13,Q19-42). The remaining 29% (12/42) are validated questionnaires (Q1-7,Q14-18). Sixty-seven percent (8/12) of the validated questionnaires have been specifically validated for RD: five of them are disease-specific (Q14-15 are CF-specific, Q17 is for pulmonary arterial hypertension, Q18 for young people with inflammatory bowel disease and Q16 for paediatric cancer) and three have been validated for multiple chronic diseases, including CF (Q1–Q3).

Of note, 18 questionnaires (9 validated and 9 unvalidated) were fully available (Q1,Q3-7,Q10,Q12-14,Q17-18,Q27,Q30,Q35-36,Q40,Q42), 17 questionnaires (3 validated and 14 unvalidated) were only partially available (i.e. not all questions or items were disclosed in the paper or made available in other sources) (Q2,Q9,Q11,Q15-16,Q19-20,Q25-26,Q28,Q31-34,Q37,Q39,Q41) and 7 (all unvalidated) were unavailable, with very little to no information on the questions being accessible (Q8,Q21-24,Q29,Q38).

The number of domains and categories covered in each questionnaire is detailed Table 6. The CF Patient Experience and Satisfaction with Care Services (Q14) is the only questionnaire covering all 11 domains [27–30], and it incorporates the highest number of categories (31/71). In second place comes the YHC-SUN (Q1) [36] with 9 domains and 30 categories, followed by the CHC-SUN (Q2) [34, 35] with 9 domains and 25 categories (these two questionnaires lack the ‘Trust’ and ‘Safety’ domains). All three are validated questionnaires: the first one targets CF adult patients while the other two are directed at adolescents or parents of children living with a chronic disease. Domain and category distribution across all questionnaires is described in detail in Additional file 4. It is similar to the distribution observed in the papers as the three most covered domains are ‘People-centredness’ (88%, 37/42), ‘Comprehensiveness’ (79%, 33/42) and ‘Self-management support’ (79%, 33/42); and the three least commonly assessed domains are ‘’Service aspects of care’ (24%, 10/42), ‘Trust’ and ‘Safety’ (12%, 5/42 each). The domain ‘Diagnosis & Treatment/Interventions’ is mostly present in unvalidated questionnaires (53% unvalidated vs 42% validated), while all other domains are more represented in validated questionnaires.

**Table 6:** Patient experience with care questionnaires identified and included (*n* = 42 questionnaires)

Questionnaire Reference (No)	Focus	Full validation or no/partial validation	Available in papers^1^	Nb of domains and categories covered
Q1. **YHC-SUN - Youth Healthcare measure-satisfaction, utilization, and needs** Developed and validated by: [36] Applied by [52]:	Generic	**Full** Over 30 rare diseases including CF*	Fully	9/11 domains 30/71 categories
Q2. **CHC-SUN - Child Health Care Questionnaire - Satisfaction, Utilization and Needs** Developed and validated by: [34, 35] Applied by: [53] Adapted by: [36]	Generic	**Full** including for CF*	Partially	9/11 domains 25/71 categories
Q3. **Health Care Preferences of Chronically Ill Adolescents** Developed and validated by: [56] Applied by: [28]	Generic	**Full** CF*, IBD, JRA	Fully	9/11 domains 16/71 categories
Q4. **GCSS - Genetic Counselling Satisfaction Scale** Developed and validated by: [90] Applied by: [43]	Generic	**Full** Some rare diseases	Fully	5/11 domains 5/71 categories
Q5. **SCNS-SF34 - Supportive Care Needs Survey Short Form** Developed and validated by: [91] Applied by: [75]	Generic	**Full**	Fully	4/11 domains 10/71 categories
Q6. **Family Experiences with Coordination of Care Survey** Developed and validated by: [92] Applied by: [45]	Generic	**Full** Not for rare diseases	Fully	5/11 domains 17/71 categories
Q7. **CSQ-4 - Customer satisfaction questionnaire** Developed and validated by: [93] Applied by: [52]	Generic	**Full** Not for rare diseases	Fully	1/11 domains 2/71 categories
Q8. **Patient experiences of transition from paediatric to adult services** Developed and applied by: [40]	Generic	No/partial Tested in IBD, and CF*	No	5/11 domains 10/71 categories
Q9. **Determinants of Quality of Care for Adolescents and Young Adults With Chronic Illnesses** Developed and applied by: [42]	Generic	No/partial Applied in CF*	Partially	5/11 domains 15/71 categories
Q10.** IEXPAC-rare-diseases** Developed and applied by: [44]	Generic (rare diseases)	No/partial	Fully	7/11 domains 21/71 categories
Q11. **Rare disease patient experiences of health service support and mental health** Developed and applied by: [50]	Generic (rare diseases)	No/partial	Partially	7/11 domains 15/71 categories
Q12. **Value Of The Complex Care Service** Developed and applied by: [37]	Generic (rare diseases)	No/partial	Fully	7/11 domains 13/71 categories
Q13. **Experience of healthcare at reference centres of patients and parents of children with rare conditions** Developed and applied by: [46]	Generic (rare diseases)	No/partial	Fully	6/11 domains 14/71 categories
Q14. **CF patient experience and satisfaction with care services** Developed, validated and applied by: [12, 17–19]	CF*	Full	Fully	11/11 domains 31/71 categories
Q15. **CF patient and family experience of care survey** Developed and validated by: [20] Applied by: [21]	CF*	Full	Partially	7/11 domains 16/71 categories
Q16. Clinic satisfaction among adult survivors of childhood cancer* (2 questionnaire administered at different timepoints) Developed and validated by: [15] Applied by: [16]	Paediatric cancer	Full	Partially	7/11 domains 17/71 categories
Q17. **PAHC-PREM: Patient’s Experiences of Health Care in Pulmonary Arterial Hypertension Outpatient Clinics** Developed, validated and applied by: [22]	PAH	Full	Fully	5/11 domains 9/71 categories
Q18. **Satisfaction with care in young persons with Inflammatory bowel disease (IBD)** Developed, validated and applied by: [23]	IBD	Full	Fully	9/11 domains 25/71 categories
Q19. **ACE: Adult Cystic fibrosis Experience Score** Developed and applied by: [30]	CF*	No/partial	Partially	8/11 domains 11/71 categories
Q20. **Quality improvement program survey in CF care** Developed and applied by: [48]	CF*	No/partial	Partially	7/11 domains 11/71 categories
Q21. **CF Trust Standards of care** Developed and applied by: [34]	CF*	No/partial	No	7/11 domains 11/71 categories
Q22. **Expectations and needs of CF patients and the CF center’s response** Developed and applied by: [38]	CF*	No/partial	No	6/11 domains 8/71 categories
Q23. **CF adherence promotion and satisfaction with care** Developed and applied by: [32]	CF*	No/partial	No	5/11 domains 7/71 categories
Q24. **CF Ward Round Survey** Developed and applied by: [35]	CF*	No/partial	No	3/11 domains 6/71 categories
Q25. **Disease Experiences of Minority Adults With CF** Developed and applied by [36]:	CF*	No/partial	Partially	3/11 domains 4/71 categories
Q26. **CF related adaptation of the Foundation Trusts Friends and Family Test** Developed and applied by: [33]	CF*	No/partial	Partially	2/11 domains 4/71 categories
Q27. **Partnership between parents of children with CF and the CF team** Developed and applied by: [72]	CF*	No/partial	Fully	1/11 domains 3/71 categories
Q28. **End-of-Life Care Quality for Children With Cancer** Developed and applied by: [37]	Paediatric cancer	No/partial	Partially	5/11 domains 12/71 categories
Q29. **Survey on high quality, patient centred and coordinated care for Alstrom syndrome** Developed and applied by: [54]	Alstrom syndrome	No/partial	No	9/11 domains 23/71 categories
Q30. **Poland syndrome patient experiences with diagnosis and care** Developed and applied by: [46]	Poland syndrome	No/partial	Fully	9/11 domains 14/11 categories
Q31. **Systemic sclerosis (SS) patient experience with healthcare and perceived care quality** Developed and applied by: [51–53]	SS*	No/partial	Partially	8/11 domains 19/71 categories
Q32. **Quality of Care of Patients with Chronic Lymphoedema** Developed and applied by: [50]	Chronic lymphoedema, incl; primary lymphoedema	No/partial	Partially	7/11 domains 13/71 categories
Q33. **Physical, emotional and financial impact of myelofibrosis** Developed and applied by: [45]	Myelofibrosis*	No/partial	Partially	7/11 domains 11/71 categories
Q34. **Maternal Reports of Satisfaction With Care and Outcomes for Children With Craniosynostosis** Developed and applied by: [40]	Craniosynostosis*	No/partial	Partially	7/11 domains 11/71 categories
Q35. **Haemophilia Treatment Center (HTC) - National Patient Satisfaction Survey** Developed and applied by: [39]	Haemophilia, VWD*	No/partial	Fully	6/11 domains 13/71 categories
Q36. **Mountain States Regional Haemophilia and Thrombosis Centre Patient Satisfaction Survey** Developed and applied by: [41]	Haemophilia A and B, VWD*	No/partial	Fully	6/11 domains 13/71 categories
Q37. **Quality of care for multiple endocrine neoplasia disorders** Developed and applied by: [49]	MEN	No/partial	Partially	6/11 domains 7/71 categories
Q38. **Parental experience of, and satisfaction with, our tertiary paediatric service for children with Non-cystic fibrosis bronchiectasis (NCFB) and primary ciliary dyskinesia (PCD)** Developed and applied by: [44]	NCFB, PCD	No/partial	No	5/11 domains 8/71 categories
Q39. Primary Biliary Cholangitis (PBC) patient reported experiences * app-based PREMs Developed and applied by: [47]	PBC*	No/partial	Partially	4/11 domains 6/71 categories
Q40. **Paediatric hereditary angioedema patient experience survey** Developed and applied by: [42]	Paediatric hereditary angioedema	No/partial	Fully	3/11 domains 5/71 categories
Q41. **Patient experience with healthcare transition from childhood to adulthood in pseudoxanthoma elasticum** Developed and applied by: [43]	Pseudoxanthoma elasticum	No/partial	Partially	3/11 domains 4/71 categories
Q42. **Alkaptonuria patient experiences of receiving diagnosis and care** Developed and applied by: [31]	Alkaptonuria	No/partial	Fully	3/11 domains 3/71 categories

^1^Available in papers: Fully = questionnaire was fully available; Partially = questionnaire was partially available (i.e. not all questions or items were disclosed in the paper or made available in other sources), No = questionnaire was not available

Disease-specific keywords for these diseases were included in the literature search string used to query the databases (Additional file 1)

CF*=Cystic Fibrosis, IBD = Inflammatory Bowel Disease, JRA = Juvenile Rheumatoid Arthritis, MEN = Multiple Endocrine Neoplasia disorders, NCFB = Non-cystic fibrosis bronchiectasis, PAH = Pulmonary Arterial Hypertension, PBC*=Primary Biliary Cholangitis, PCD = primary ciliary dyskinesia, SS*=Systemic Sclerosis, VWD*=Von Willebrand Disease

## Discussion

### Proposing a first structured definition of patient and carer-reported experience with secondary and/or tertiary care for PLWRD

This study maps out the published evidence on the experience of PLWRD and family carers with secondary and tertiary healthcare, proposing a first definition of this concept by structuring it into 11 domains: ‘Access’, ‘Comprehensiveness’, ‘Continuity’, ‘Coordination’, ‘People-centredness’, ‘Safety’, ‘Self-management support’, ‘Trust’, ‘Overall perceived quality of care’, ‘Diagnosis & Treatment/Interventions’ and ‘Service Aspects of Care’. These domains were further defined through 71 author-built categories. Nine of the 11 domains used in this definition came from the PREM branch of the OECD PaRIS study [25, 26]. They were initially outlined into 42 author-built categories and used to streamline data extraction. The PaRIS framework was selected because it pursues goals that are relevant for PLWRD across Europe. It aims to monitor and improve the healthcare experience of people living with a wide range of complex, chronic conditions across countries, using it to help policy makers and all relevant stakeholders “identify best practices, fuel international learning and foster a dialogue with patients and service providers about how to further improve the performance and people-centredness” of care services [25]. These aspirations of the PaRIS framework imprint reliability and acceptability to a healthcare experience framework for PLWRD [26, 94]. Nevertheless, the PaRIS study aims to monitor and improve the primary care experience of people over 45 years-old living with multiple chronic conditions. During data extraction and while processing the published evidence, we identified the need to add two new domains (‘Diagnosis & Treatment/Interventions’ and ‘Service Aspects of Care’) and 29 additional categories to better capture and report the evidence found in the literature on RD in secondary and tertiary care. Access to timely diagnosis and/or treatment is a long-standing and specific unmet need of PLWRD [5, 12, 95–98] which is reflected in these findings.

### Importance of a validated PREM to measure and improve patient and carer-reported experience with secondary and tertiary care for PLWRD

None of the 61 papers nor any of the 42 questionnaires included in this review fully covered all the healthcare experience domains and categories. The CF Patient Experience and Satisfaction with Care Services questionnaire was the only questionnaire covering all 11 domains. Still, it was developed for one RD and only covered 44% (31/71) of the healthcare experience categories mapped in this review. Contrary to what is already available in other large and diverse populations, such as in chronic diseases and primary care [99–101], none of the PREMs identified were fully validated across RDs.

PREM validation creates a virtuous circle [102, 103], as it ensures questionnaire reliability, systematic high-quality result collection and promotes PREM endorsement by international organisations, such as the OECD [26, 94] and the European Commission [104], thereby promoting PREM uptake and implementation in clinical practice and settings. This approach enables more robust, rigorous, and comprehensive data collection, facilitating meaningful comparisons across healthcare units, countries, regions, and diseases. However, the validation of PREMs should be guided by a well-defined conceptual framework. This study offers valuable insights for future research focused on finalising a conceptual framework of healthcare experiences in secondary and tertiary care for PLWRD and their family carers, and on validating generic RD-PREMs to capture this experience.

### Study strengths and limitations

The adopted review methodology enabled us to [1] pinpoint relevant concepts, domains and categories related to RD patients and family carers’ experience with secondary and tertiary healthcare [2]; identify existing instruments to assess PLWRD experience with healthcare, as well as [3]; explore the differences between qualitative, quantitative, and mixed methods studies.

(1) This literature review was restricted to secondary and tertiary healthcare settings and did not include studies focusing only on primary care. This strategic choice relies on published evidence on the positive impact that having access to and receiving care at hospital units specialised in RDs has on the healthcare experiences of patients and family caregivers [9–11], making access to high-quality secondary and tertiary care a clinical priority for PLWRD. It also potentiates better validation of future PREMs, since the combined approach of face-to-face PREMs distribution in specialised units [9–11] in addition to online dissemination through patient associations yields a better response rate [105, 106]. However, this does not imply that primary care, and especially care coordination between primary care and other care settings, should not be further explored and included in the conceptual framework and resulting PREMs.

(2) Some of the limitations of this study are inherent to the exploratory nature of the scoping review methodology, which was constrained by the availability of peer-reviewed abstracts and full-text papers, and by a partial access to some of the applied questionnaires.

(3) Qualitative papers are underrepresented in our study, which may result from a publication bias, i.e., researchers facing greater difficulties in publishing qualitative results [107, 108]. However, showcasing the added value of taking a more inclusive approach, some healthcare experience domains, such as ‘Trust’, ‘Diagnosis & Treatment/Interventions’, as well as many of the categories of the ‘Comprehensiveness’ and ‘Coordination’ domains were often restricted to or more present in qualitative papers.

### Next steps and recommendations to finalise a framework of healthcare experience with secondary and tertiary care for PLWRD and their family carers, and to validate generic RD PREMs

This literature review lays the foundations for further research ensuring the robust assessment of the healthcare experience of PLWRD and their family carers in secondary and tertiary care. PREM development includes further steps such as [1] finalising a conceptual framework of healthcare experience with secondary and tertiary care for PLWRD through a multi-stakeholder consultation to discuss the first structured definition developed in this study; and [2] developing and validating generic RD PREMs. Below are 5 guiding recommendations based on the evidence generated by this review.

*Recommendation 1: Involve multiple stakeholders*. Multi-stakeholder consultations are key for multi-faceted topics such as healthcare experience [34–36]. Harnessing the experiences and perspectives of PLWRD and family carers’ on the domains and categories identified through this literature review is pivotal to ensure that the final framework and PREMs reflect and represent their needs and priorities. Some domains and categories listed in this review were restricted or predominantly reported by qualitative papers using focus groups, interviews with PLWRD and their relatives [35, 77, 80, 83, 88], pointing out the importance of thoroughly listening to these stakeholders. HCPs and other relevant healthcare actors, such as hospital managers and healthcare quality experts, should also be heard. PLWRD, their family members and professional stakeholders can (i) provide their informed views on the conceptual framework domains and categories; (ii) share insights on the feasibility and practicality of implementing a RD PREM; (iii) guide the on-site PREM administration across healthcare centres, countries, and ERNs; (iv) contribute to ensuring that the PREMs are effectively implemented to improve healthcare’s clinical responsiveness and cost-effectiveness.

*Recommendation 2: Ensure RD diversity.* While there are over 6000 known RD, this literature review only covers a limited number of RD and RD groupings, accounting for less than 2% of all RD. Even if all RD cannot be represented, a diverse purposive sample could be created by considering at least: the genetic or non-genetic nature of the disease, the typical age of disease onset, its duration and progression (acute vs chronic, relapsing/remitting, degenerative vs stable), the variety of disability status as well as disease complexity, as measured by the number and types of body parts affected. All these factors can significantly impact the service, coordination and collaboration needs of PLWRD. The ERN disease coverage and distribution could also guide disease selection.

*Recommendation 3: Cater for all age groups.* While adolescents were underrepresented in the papers considered in this scoping review, 70% of RDs are estimated to have a paediatric onset [109] and adolescents living with a RD have been found to experience specific challenges, such as longer diagnostic odysseys [110] or specific care barriers and enablers linked to self-management, empowerment, and school-interventions [111]. The literature on PREMs for adolescents also shows that they are willing to evaluate their care themselves. Hence, future steps should foresee the creation of at least two self-reported versions: one for adult patients, and one for adolescent patients. A proxy questionnaire for family carers answering for children or adults living with a RD, especially if patients have an intellectual disability should also be considered. A proxy questionnaire should include specific questions for carers such as their own access to emotional support, which was one of the lowest scores in a pilot survey on PLWRD [9–11].

*Recommendation 4: Consider gender and ethnicity.* Ensuring a well-balanced gender distribution is crucial, since gender has been found to impact the healthcare experience of PLWRD [112–115]. Ethnicity was noticeably absent from most of the papers in this literature review, especially in European countries other than the UK. This may derive from ethnicity information collection being perceived as a sensitive and subjective topic in many countries, particularly in Europe [116]. Some studies have shown healthcare inequities linked to ethnicity with ethnic minorities assessing their healthcare experiences with lower scores, including in RD [65, 117, 118]. Several national and international initiatives, including the PaRIS study, are advocating for and/or providing guidance on systematic and clear ethnicity data collection to study and address the unmet healthcare needs of underprivileged ethnic groups [119, 120]. Hence, it should be carefully considered whether and how ethnicity should be captured and used both in the consultation process and in the PREMs.

*Recommendation 5: Ensure a diverse geographic coverage.* Country representation is also important to consider, as health systems are not equally prepared to manage and support PLWRD and their family carers. The services offered widely differ from country to country. For instance, regarding access to diagnosis and time to diagnosis, or access to specialised care referrals and dedicated facilities [95–98]. To overcome any potential biases emerging from this country overrepresentation, considering cross-cultural and multi-lingual adaptations of the PREMs is critical.

## Conclusion

This scoping review mapped the domains and categories of healthcare experience with secondary and tertiary care for PLWRD and their family carers, serving as an informative basis for the future development of a healthcare experience framework for RD. This study also lays the foundations for creating generic RD-PREMs which can inform policy decisions and quality improvement initiatives at the hospital, regional, country, and European levels. These efforts will contribute to creating a more patient-centred healthcare environment that promotes equity and enhances quality of care for all PLWRD and their carers, regardless of age, gender, ethnicity, disease or geographic location.

## Supplementary Information

Below is the link to the electronic supplementary material.

Supplementary Material 1

Supplementary Material 2

Supplementary Material 3

Supplementary Material 4

## Data Availability

All data generated or analysed during this study are included in this published article and its supplementary information files.
