# Supplementary material for: Patient-reported experiences of people living with a rare disease with secondary and tertiary care: a scoping review
Source: Orphanet J Rare Dis. 2026 Nov 4;21:388. doi: 10.1186/s13023-026-04368-5 (PMC13733059; doi:10.1186/s13023-026-04368-5)
Supplement: Supplementary file 1 [file 13023_2026_4368_MOESM1_ESM.docx]

**Additional file 1. Medline Search strategy and keywords**

Date of final search: 25 September 2022.

Papers published between January 2005 and September 2022 were considered.

**Medline**

Search 1:

("rare disease*" or "rare condition*" or "complex disease*" or "complex condition*") AND ("patient experience" or “PREM” or "healthcare quality" or "care quality" or "quality of care” or “satisfaction with care”)

Search 2:

(“Osteogenesis imperfecta” OR Achondroplasia OR Craniosynostosis OR “Noonan syndrome” OR “Disorders of calcium and phosphate homeostasis” OR “epilepsy*” OR “glomerulopathy*” OR “Retinis Pigmentosa” OR “Achalasia” OR “Cystic Fibrosis” OR “dystonia” OR “Ehler-Danlos” OR “nevi” OR “nevus” OR “nevoid” OR “rare neoplasm” OR “rare sarcoma” OR “von Willebrand” OR “thalassaemia” OR “Myeloproliferative neoplasm” OR “cystinuria” OR “Hirschprung disease” OR “neuropathy*” OR “Neurofibromatos*” OR “Long QT syndrome” OR “familial cardiomyopathy” OR “RASopathy” OR “Cohesinopathy” OR “Lysosomal storage disorders” OR “paediatric cancer” OR “Primary Biliary Cholangitis” OR “Primary Biliary Cirrhosis” OR “Vascular Liver Disease” OR “Systemic sclerosis” OR “primary immunodeficiency” OR “paediatric organ transplantation” OR “Hereditary Haemorrhagic Telangiectasia”) AND ("patient experience" or “PREM” or "healthcare quality" or "care quality" or "quality of care“ or “satisfaction with care”)

**EMBASE**

Search 1:

(rare-disease* or rare-condition* or complex-disease* or complex-condition*).mp. AND (patient-experience or PREM or healthcare-quality or care-quality or quality-of- care or satisfaction-with-care).mp.

Search 2:

(Osteogenesis imperfecta OR Achondroplasia OR Craniosynostosis OR Noonan syndrome OR Disorders of calcium and phosphate homeostasis OR epilepsy* OR glomerulopathy* OR Retinis Pigmentosa OR Achalasia OR Cystic Fibrosis OR dystonia OR Ehler-Danlos OR nevi OR nevus OR nevoid OR rare neoplasm OR rare sarcoma OR von Willebrand OR thalassaemia OR Myeloproliferative neoplasm OR cystinuria OR Hirschprung disease OR neuropathy* OR Neurofibromatos* OR Long QT syndrome OR familial cardiomyopathy OR RASopathy OR Cohesinopathy OR Lysosomal storage disorders OR paediatric cancer OR Primary Biliary Cholangitis OR Primary Biliary Cirrhosis OR Vascular Liver Disease OR Systemic sclerosis OR primary immunodeficiency OR paediatric organ transplantation OR Hereditary Haemorrhagic Telangiectasia).mp AND (patient experience or PREM or healthcare quality or care quality or quality of care or satisfaction with care).mp

**CINAHL**

Search 1:

("rare disease*" or "rare condition*" or "complex disease*" or "complex condition*") AND ("patient experience" or “PREM” or "healthcare quality" or "care quality" or "quality of care“ or “satisfaction with care”)

Search 2:

(“Osteogenesis imperfecta” OR Achondroplasia OR Craniosynostosis OR “Noonan syndrome” OR “Disorders of calcium and phosphate homeostasis” OR “epilepsy*” OR “glomerulopathy*” OR “Retinis Pigmentosa” OR “Achalasia” OR “Cystic Fibrosis” OR “dystonia” OR “Ehler-Danlos” OR “nevi” OR “nevus” OR “nevoid” OR “rare neoplasm” OR “rare sarcoma” OR “von Willebrand” OR “thalassaemia” OR “Myeloproliferative neoplasm” OR “cystinuria” OR “Hirschprung disease” OR “neuropathy*” OR “Neurofibromatos*” OR “Long QT syndrome” OR “familial cardiomyopathy” OR “RASopathy” OR “Cohesinopathy” OR “Lysosomal storage disorders” OR “paediatric cancer” OR “Primary Biliary Cholangitis” OR “Primary Biliary Cirrhosis” OR “Vascular Liver Disease” OR “Systemic sclerosis” OR “primary immunodeficiency” OR “paediatric organ transplantation” OR “Hereditary Haemorrhagic Telangiectasia”) AND ("patient experience" or “PREM” or "healthcare quality" or "care quality" or "quality of care“ or “satisfaction with care”)
