# Supplementary material for: Patient-reported experiences of people living with a rare disease with secondary and tertiary care: a scoping review
Source: Orphanet J Rare Dis. 2026 Nov 4;21:388. doi: 10.1186/s13023-026-04368-5 (PMC13733059; doi:10.1186/s13023-026-04368-5)
Supplement: Supplementary file 2 [file 13023_2026_4368_MOESM2_ESM.docx]

**Additional file 2. Data extraction template.** Both closed-ended and open data extraction fields were included for completeness.

The data extraction template was further organised in 3 main sections:

- **Section 1** corresponds to general information about the paper
- **Section 2** to the methods used, being further sub-divided into **section 2.1** quantitative and **section 2.2** qualitative research.
- **Section 3** lists the domains and categories of healthcare experience). Originally, only the 9 domains of the PaRIS study and 42 categories (in black) were considered. As data extraction and analysis were an iterative process and allowed for qualitative data collection, two additional domains and 29 categories were assigned to the domains at a later stage (in green).

|  | Data extraction fields | |
| --- | --- | --- |
| Section | Open fields | Closed-ended fields |
| Section 1 - General information | Authors, Publication year, Title, Notes | Country(ies) where the study was conducted:  *Answer options*: US, UK, Canada, Australia, Netherlands, Other |
| Section 2 – Methods | Aim of study, Disease name/disease group, Comments/Notes on methods | Participants  *Answer options:* Patients, Carers, Other  Age of patients participating in the study  *Answer options:* < 18 y.o., 18-65 y.o., 65 + y.o.  Study design  *Answer options:*   - Systematic review - Quantitative research - Qualitative research - Mixed methods - Longitudinal/repeated measures - Evaluation of intervention - Case report - Other |
| Section 2.1. – Quantitative research | Name of the scale used (if any), Number of participants (effective sample size after non-response and drop-out), Response rate | Quantitative research  *Answer options*:   - Validation activities for the questionnaire used in the study - Using existing validated scale, - Sensitivity analysis to examine robustness of results - Multivariate analysis to adjust for potential confounders - Other |
| Section 2.2. – Qualitative research | Number of participants (effective sample size after non-response and drop-out), Response rate | Qualitative research  *Answer options*:   - Purposive sampling - Convenient sampling - Multiple phases for qualitative analysis, - Enhancing credibility of results (multiple researchers, checking with interviewees) - Other |
| Section 3 – Domains of patient experience with care (PaRIS study, OECD)  Additional rare disease-specific domains of patient experience with secondary and tertiary healthcare | **NA** | Domains taken into account  *Answer options*:   - Access - Comprehensiveness - Continuity - Coordination - Safety - People-centredness - Self-management support - Trust - Overall perceived quality of care - Service aspects of care - Diagnosis & Treatment/Interventions - Other   **Access**  *Answer options*:   - Timing of appointments – suitability - How the appointment was made (in person, by phone, online…) – easiness, - Location of appointments – convenience - Mode of appointment (in person, phone, video call) – acceptability - Duration of appointments (little time/enough time) - **Waiting time** (in the waiting room, to get an appointment after doing tests, outside of routine visits, unexpected cancellations) **Insurance coverage and financial burden** (parking, travelling, testing costs, access other types of financial support and reimbursement of care) - Other   **Comprehensiveness**  *Answer options*:   - Access to multidisciplinary care - Questions on the patient's health habits - Healthcare professionals asked about the impact of the disease on patient's / carer's life - Guidance on procedures for disability status and fiscal benefits - Guidance about social services and education resources - Psychological/emotional support (patient and/or carer) - Involvement of/access to social worker - Peer-support (patient organisation or other): existing peer-support, access to peer-support - **Access to specialized providers** - **Supply/access to medical/physical aid equipment, home care** - **Care adapted to the evolving patient needs** (related to age, independent living, starting a relationship, starting a job) **Access to/existence of clinical care guidelines and necessary/optimal testing/monitoring Liaison and collaboration with schools and school-related services** (awareness, liaising with school nurses, learning interventions, systematic instructions in school by HCPs) **Respite care and bereavement support** (for family carers) - Other   **Continuity**  *Answer options*:   - Care continuity - Is there a named healthcare professional who they can contact / how contactable are they - Have an alert card about their condition which can be used in emergency situations / when they are in contact with a new healthcare professional - Follow - up path planned after the visit/hospital discharge - Follow-up after visit (including booking of regular examinations...) - **Access to and satisfaction with transition of pediatric to adult care Access to and satisfaction with emergency care** - Other   **Coordination**  *Answer options*:   - Existence of a case manager / care coordinator - Effectiveness of how care is coordinated - Transfer of information to the local doctor: specialist / primary care central/ GP - All specialists needed to address the case were present during consultations - Team working of the healthcare professionals involved in care - Given a treatment plan (written / digital) - **Access to medical records/ history/ registries** - Other   **Safety**  *Answer options:*   - **Infection prevention measures**, e.g. hand hygiene **Safety of care and of care spaces** (including patient segregation if/when necessary) - Other   **People-centredness**  *Answer options*:   - HCP respect - HCP communication/listening skills, - Patient/carer participation/involvement in care decision-making - Preferences of patient taken into account / involved in decision-making - Other   **Self-management support**  *Answer options*:   - Information on where to turn with questions (person / website / support group) - Information on treatment (clear info on objectives, results...) - Peer-support (patient organisation or other): informed of availability, given contact - Information on reimbursement of care - Information provided on the rare condition (before /during / after diagnosis) - Information on local therapy, rehab, physio facilities - **Information on tests and test results** - **Information on elective admissions and upon hospital discharge** - **Information on research and registries Information on everyday management and daily life** (sex life, sports) - **Education/self management programmes Information for carers and family members Quality of the information provided** (accessibility, effectiveness / accuracy, timeliness, understandability, format) - Other   **Trust**  *Answer options*:   - Trust / confidence in the care team - Other   **Overall perceived quality of care**  *Answer options*:   - Technical/Instrumental quality of care: Perceived skills of the care team (including knowledge levels / expertise of the healthcare professionals providing care) - **Patient participation in research Overall satisfaction with care Overall experience with the care received** - Other   **Service aspects of care**   - Premises/ Facilities (cleanliness, toilets, seating, room design and furnishing, functionality of care spaces, play areas, availability of food/snacks) - Food Friendliness of support staff Received reminders for appointments - Accommodation for close family - **Toys provided** - **Administrative aspects of care**   **Diagnosis & Treatment/Interventions**   - **Discomfort in pre-diagnosis phase of care** - **Access to appropriate diagnostic testing** (e.g. genetic testing) - **Diagnosis** (timely, misdiagnosis, length of diagnosis journey, emotional impact, feeling dismissed) - **Access to treatment** (medication refills, emergency medication, inpatient treatment, home treatment, local hospital, bureaucracy) - **Treatment safety** (e.g long-time/severe side effects and/or adverse reactions) - **Treatment effectiveness and satisfaction** (existence and/or access to guidelines or other validated tools to assess and monitor response) - **Adherence to treatment** (e.g. discussing/addressing barriers to adherence) |
