# Supplementary material for: Patient-reported experiences of people living with a rare disease with secondary and tertiary care: a scoping review
Source: Orphanet J Rare Dis. 2026 Nov 4;21:388. doi: 10.1186/s13023-026-04368-5 (PMC13733059; doi:10.1186/s13023-026-04368-5)
Supplement: Supplementary file 3 [file 13023_2026_4368_MOESM3_ESM.docx]

**Additional file 3.** Table of study main characteristics

| **I. Author, location, date of publication, method/instrument** | **II. Participant groups (age of patients); disease/disease group** | **III. Number participants per group (response rate)** | **IV. Validation and patient involvement measures / activities** |
| --- | --- | --- | --- |
| **Quantitative papers** | | | |
| Husson 2021 (1);  US, UK, Canada, Australia, Netherlands, Germany, Belgium, China, Greece, Brazil, Denmark, Ecuador, Estonia, France, Italy, Macedonia, Poland, Portugal, Turkey;  Questionnaire | Patients (18-65 y.o.; 65+ y.o.);  Epithelioid Haemangioendothelioma | 115 (NA) | **Existing validated scale(s)**: Supportive Care Needs Survey Short Form, SCNS-SF34) (2).  **Patient involvement:** Participant recruitment done though patient organisation networks; Patient-initiated study. |
| Steinkamp 2015 (3);  Europe: Germany;  Questionnaire | Patients (<18 y.o.; 18-65 y.o.), carers;  Cystic Fibrosis (CF) | 1221 adults with CF (75%); 891 parents of patients with CF (74%). | **Existing validated scale(s):** generic items from the Picker Patient Experience Questionnaire(4);  **Other validation activities**: focus groups with CF adult patients and parents of children with CF; Interviews with professional experts.  **Patient involvement:** Patients and carers reviewed and pretested the questionnaire in 4 centres. |
| Ellemunter 2015 (5);  Europe: Germany;  Questionnaire | Patients (<18 y.o.; 18-65 y.o.), carers;  Cystic fibrosis (CF) | 2009: 31 parents (73%), 32 adults (84%), 13 adolescents (72%).  2011: 139 parents and adults (80% parents; 77% adults), 26 adolescents (81%) | **Existing validated scale(s):** generic items from the Picker Patient Experience Questionnaire (children and adult versions); for adolescents the Health Care Preferences of Chronically Ill Adolescents scale developed and validated by (6). **Other validation activities**: focus groups with CF adult patients and parents of children with CF; interviews with professional experts.  **Patient involvement:** Patients and carers reviewed and pretested the questionnaire in 4 centres. |
| Cafaggi 2011 (7);  Europe: Italy;  Questionnaire | Patients (<18 y.o.; 18-65 y.o.), carers;  Cystic fibrosis (CF) | 87 (35%) | NA |
| Riekert 2012 (8);  Outside Europe: USA;  Questionnaire | Patients (<18 y.o.), carers;  Cystic fibrosis (CF) | 290 parents (NA); 290 adolescents (NA) | **Existing validated scale(s):** none.  **Other validation activities:** Cohen Kappa statistic |
| Heeres 2015 (9);  Europe: Netherlands;  Questionnaire | Carers of patients (<18 y.o.);  Cystic fibrosis (CF) | 66 (49%) | NA |
| Spierings 2020 (10);  Europe: Netherlands;  Questionnaire | Patients (18-65 y.o.);  Systemic sclerosis (SS) | 650 (31%) | **Existing validated scale(s):** items from the Consumer quality index (CQ index) for rheumatoid arthritis (11); **Other validation activities:** 3 focus groups (23 patients, 12 rheumatologists, 5 specialised nurses); questions discussed in a multi-stakeholder group (a patient representative, 3 rheumatologists and a researcher);  **Patient involvement:** Questionnaire co-developed with patients; survey tested by 5 patients. |
| Berry 2019 (12);  Europe: UK;  Questionnaire | Patients (NA), carers;  Cystic fibrosis (CF) | 2016: 23 (78%). 2017: 22 (100%) | NA |
| Riske 2020 (13);  Outside Europe: USA;  Questionnaire | Patients (<18 y.o.; 18-65 y.o.; 65+ y.o.), carers;  Haemophilia and Von Willebrand disease | 5,006 (18%) | **Existing validated scale(s):** CDC National Survey of Children with Special Healthcare Needs (14); CAHPS Measures of Patient Experience (15). **Other validation activities:** question content aligned with legacy regional surveys; question formats harmonized with nationally validated surveys (facilitate comparisons and enhance scientific robustness).  **Patient involvement:** Patients reviewed questionnaire content and the administration process. |
| Thomas 2019 (16);  Europe: UK;  Questionnaire | Patients (<18 y.o.; 18-65 y.o.);  Cystic fibrosis (CF) | 22 (100%) | NA |
| Finlayson 2020 (17);  Outside Europe: Australia;  Questionnaire | Patients (18-65 y.o.);  Cystic fibrosis (CF) | 17 (NA) | **Existing validated scale(s):** generic items from the Picker Patient Experience Questionnaire (4). **Other validation activities**: ACE score based on themes elicited from (18) another study with an adult CF satisfaction survey; 3 final questions for participants to rate the utility and ease of completing the ACE Score. |
| Mitchell-Thain 2021 (19);  Europe / outside Europe:  UK and 19 other countries (not specified);  Questionnaire | Patients (18-65 y.o.; 65+ y.o.);  Primary Biliary Cholangitis | 1^st^ app-based survey: 189 (NA). 2^nd^ app-based survey: 171 (NA). | NA |
| Sharif 2021 (20);  Europe: UK;  Questionnaire | Carers of patients (<18 y.o.;.);  Non-cystic fibrosis bronchiectasis, primary ciliary dyskinesia | 30 (29%) | NA |
| Hutchins 2021(21);  Outside Europe: USA;  Questionnaire | Patients (18-65 y.o.);  Cystic fibrosis (CF) | 82 (25%) | NA |
| van Laar 2013 (22);  Europe: UK;  Questionnaire | Patients and former patients (18-65 y.o.; 65+ y.o.);  Childhood cancers (not specified) | 143 (NA) | **Existing validated scale(s):** Clinic satisfaction among adult survivors of childhood cancer scale developed and validated by (23);  **Multivariate analysis** to adjust for potential confounders **Other validation activities:** internal consistency Cronbach alpha to assess scales' reliability. |
| Pougheon-Bertrand, 2018 (24);  Europe: France;  Questionnaire | Patients (<18 y.o.; 18-65 y.o.), carers, healthcare professionals involved in cystic fibrosis care;  Cystic fibrosis (CF) | 12 (NA) ^b16^ | **Existing validated scale(s):** none. **Validation activities**: author-built questionnaire based on the 5 dimensions of the Chronic Care Model, the patient and family engagement framework (25) and other unnamed validated instruments;  **Patient involvement:** Patients as research partners (involved in project development and questionnaire design). |
| Homa 2015 (26) ;  Outside Europe: USA;  Questionnaire | Patients (<18 y.o.; 18-65 y.o.; ; 65+ y.o.), carers;  Cystic fibrosis (CF) | 839 adults (28%); 1,251 parents (30%) | **Existing validated scale(s)**: CF patient and family experience of care survey (27); **Multivariate analysis** to adjust for potential confounders |
| Wong-Gibbons 2009 (28);  Outside Europe: USA;  Questionnaire | Carers of patients (<18 y.o.);  Craniosynostosis | 82 (56%) | NA |
| Ananth 2022 (29);  Outside Europe: USA;  Questionnaire | Carers of patients (<18 y.o.), bereaved parents, patient advocates, healthcare professionals;  Childhood cancer | 26 (81%), including 4 parents and patient advocates ^b19^ | **Existing validated scale(s):** none.  **Patient involvement:** Bereaved parents and patient advocates included in item prioritisation through a Delphi process.  Participant recruitment done through patient organisation networks. |
| Baldelli 2019 (30);  Europe: Italy;  Questionnaire | Patients (<18 y.o.; 18-65 y.o.), carers;  Poland syndrome | 57 (79%) | **Existing validated scale(s):** none. **Multivariate analysis** to adjust for potential confounders;  **Patient involvement:** The questionnaire was designed by a panel of experts on Poland syndrome who included both clinicians and patients or parents of patients. |
| Hacker 2006 (31);  Outside Europe: USA;  Questionnaire | Patients (<18 y.o.; 18-65 y.o.; 65+ y.o.), carers;  Haemophilia A and B,  Von Willebrand Disease ^a21^ | 100 parents (54%) 43 adolescents (52%) 123 adults (37%) | **Existing validated scale(s):** Patient Satisfaction Questionnaire (PSQ) (32); Parent Satisfaction Questionnaire; Patient satisfaction with pharmacy services (33). **Validation activities:** adapted questions from validated scales identified in a literature review.  **Patient involvement:** Questionnaire reviewed by 9 patients or parents of patients (topics addressed, completion time, question wording). |
| Read 2014 (34);  Europe: UK;  Questionnaire | Patients (<18 y.o.), carers;  Paediatric hereditary angioedema | 10 (83%) | NA |
| Britto 2007 (6);  Outside Europe: USA;  Questionnaire | Patients (<18 y.o.; 18-65 y.o.), carers;  Sickle cell disease, cystic fibrosis (CF), inflammatory bowel disease, juvenile rheumatoid arthritis | 155 (62%) | **Validation activities:** topics identified in focus groups with healthy and chronically ill adolescents; questionnaire items developed by a psychometrician and a physician; Cronbach alpha for internal validity.  **Multivariate analysis** to adjust for potential confounders.  **Patient involvement:** Questionnaire pilot tested by a small group of patients and/or carers. |
| Drewitz 2021 (35);  Europe: 18 EU countries. UK, Italy, Netherlands, Germany, France; 15 additional countries unspecified;  Questionnaire | Patients (<18 y.o.; 18-65 y.o.; 65+ y.o.), carers;  Multiple endocrine neoplasia (MEN) syndromes | 288 (NA): 203 MEN1 (NA), 67 MEN2a (NA), 18 MEN2b/3 (NA). | **Existing validated scale(s):** none. **Validation activities:** questionnaire developed with patients and patient advocates as the main contributors.  **Patient involvement:** Participant recruitment done through patient organisation networks. |
| Rudebeck 2020 (36);  Europe: France, Italy, Netherlands, Slovakia, Spain, UK. Outside Europe: Jordan;  Questionnaire | Patients (<18 y.o.; 18-65 y.o.; 65+ y.o.);  Alkaptonuria | 45 (38%) | **Patient involvement:** Data handling, including storage, source documentation, and quality control of the final analysis file and of completeness of data; Participant recruitment done through patient organisation networks. |
| Schmidt 2016 (37);  Europe: Germany;  Questionnaire | Patients (<18 y.o.; 18-65 y.o.);  Cystic fibrosis (CF), juvenile rheumatoid arthritis, inflammatory bowel disease, 30 other rare conditions mainly congenital and metabolic diseases ^a26^ | 182 (NA) | **Existing validated scale(s)**: Child Health Care Questionnaire - Satisfaction, Utilization and Needs (CHC-SUN) (38). **Other validation activities:** qualitative bottom-up-approach to validate the conceptual underpinnings of the instrument (focus groups, and interviews with young people with diabetes, cystic fibrosis and chronic inflammatory bowel disease). **Sensitivity analysis** to examine robustness of results.  **Patient involvement:** Cognitive debriefing with young people with diabetes, cystic fibrosis and chronic inflammatory bowel disease. |
| Chrysochoou 2019 (39);  Europe: Greece;  Questionnaire | Carers of patients (<18 y.o.);  Cystic Fibrosis (CF) | 60 (NA) | **Existing validated scale(s):** Child Health Care Questionnaire - Satisfaction, Utilization and Needs (CHC-SUN) (38). |
| Thyen 2018 (40);  Europe: UK, Netherlands, Germany, France, Poland, Sweden;  Questionnaire | Patients (<18 y.o.; 18-65 y.o.);  Disorders/differences of sex development (DSD): Turner Syndrome, Klinefelter Syndrome, 46, XX congenital adrenal hyperplasia, XY-DSD | 948 (31%): 261 with Turner Syndrome (NA); 173 with Klinefelter Syndrome (NA); 190 with 46, XX congenital adrenal hyperplasia (NA); 257 with XY-DSD (NA) | **Existing validated scale(s):** Customer satisfaction questionnaire (CSQ-4)(41) and an adapted version of the Youth Health Care Satisfaction, Utilization & Needs (YHC-SUN-SF)(37).  **Other validation activities:** Discriminatory power tests to decide which items to include/exclude, medical interview, retrospective chart review, medical examinations.  **Multivariate analysis** to adjust for potential confounders |
| Schmidt, 2007 (38);  Europe: UK, Netherlands, Germany, Greece, Austria, France, Sweden;  Questionnaire | Carers of patients (<18 y.o.);  Cystic fibrosis (CF) and juvenile rheumatoid arthritis ^a29^ | Pilot testing: 400 (NA) including 29 with CF (NA). Field testing/validation: 795 (NA) including 23 with CF (NA). | **Existing validated scale(s):** none.  **Other validation activities:** literature review of conceptual frameworks and existing measures; focus groups with patients and parents to generate items valid for the population; psychometric evaluation and item reduction, field, psychometric property, and validity testing of the final version.  **Sensitivity analysis** to examine robustness of results.  **Patient involvement:** Patients and parents included in an item pool creation through a card-sorting procedure and expert rating. Pilot testing by patients in all countries. |
| Sadlo 2014 (42);  Europe: Germany;  Questionnaire | Patients (<18 y.o.; 18-65 y.o.);  Inflammatory bowel disease (IBD) | 2nd item reduction survey: 86 (42%). Pilot testing: 8 patients (NA), 30 healthy volunteers (NA). Validation survey: 141 (94%). | **Existing validated scale(s):** none.  **Other validation activities:** Items constructed based on a qualitative patient internet survey, a short qualitative physician survey and a literature search; Instrument constructed in analogy to the Quality of Care Through the Patient's Eyes (QUOTE) IBD and QPP questionnaires; Internal consistency measured by calculating Cronbach’s alpha, Cohen’s kappa calculation; qualitative survey.  **Sensitivity analysis** to examine robustness of results.  **Patient involvement:** Pilot testing with IBD patients. |
| Spencer-Tansley, 2022 (43);  Europe: UK;  Questionnaire | Patients (<18 y.o.; 18-65 y.o.; 65+ y.o.), carers;  Several RDs, most represented: immunological, neurological, connective tissue, neuromuscular and endocrine conditions; most represented among carers: congenital malformations and intellectual disability (including undiagnosed conditions with a suspected genetic origin, and chromosomal abnormalities) | 913 patients (69%), 340 carers (60%). | **Existing validated scale(s):** none.  **Other validation activities**: Survey developed based on scoping review and 16 interviews with patients, carers, and patient advocates.  **Patient involvement:** Pilot testing with patients, carers and staff members of Genetic Alliance UK. Participant recruitment done though patient organisation networks. |
| Lechevalier, 2022 (44);  Europe: France;  Questionnaire | Patients (18-65 y.o.);  Pseudoxanthoma elasticum | 11 (58%) | NA |
| Waldreus, 2019 (45);  Europe: Sweden;  Questionnaire | Patients (18-65 y.o.; 65+ y.o.);  Pulmonary Arterial Hypertension | 156 (77 %) | **Existing validated scale(s):** none.  **Other validation activities:** Patient interviews were conducted.  **Sensitivity analysis** to examine robustness of results;  **Multivariate analysis** to adjust for potential confounders; Scale reliability calculated with coefficient alpha.  **Patient involvement:** Priority setting exercise with 21 patients to select the final items. |
| Herberger 2013 (46);  Europe: Germany  Questionnaire | Patients (18-65 y.o.; 65+ y.o.);  Primary lymphoedema^a34^ | 301 (86%) | NA |
| Hytiris 2021 (47);  Europe: UK;  Questionnaire | Patients (<18 y.o.), carers;  Several RDs: cardiovascular, endocrine, ENT, gastrointestinal, haematological/immune, metabolic, musculoskeletal/ dermatological, neurodevelopmental, neuromuscular, oncological, eye, renal, respiratory, other diseases, mostly syndromic conditions. | 130 (NA) | **Existing validated scale(s):** none.  **Patient involvement:** The questionnaire was developed based on the input from the Patient Advisory Group, the Steering Committee and external organisations including Genetic Alliance UK. |
| deJong 2021 (48);  Outside Europe: USA;  Questionnaire | Carers of patients (<18 y.o.; 18-65 y.o.);  Inflammatory Bowel Disease (IBD) | 101 (37%) | **Validated scale(s):** Family Experiences with Coordination of Care (FECC)(49).  **Multivariate analysis** to adjust for potential confounders |
| **Qualitative papers** | | | |
| Guilabert 2021(50);  Europe: Spain;  Questionnaire | Patients (<18 y.o.; 18-65 y.o.), carers;  50 different rare diseases, including Addison's disease, Antiphospholipid syndrome, Ehlers-Danlos syndrome, Scleroderma. | 232 adults (89%), 29 carers (11%). | **Existing validated scale:** Instrument for Evaluation of the Experience of Chronic Patients (IEXPAC) (51).  **Other validation activities:** Scale adaptation was done by a selection of specialists working on rare diseases and by applying the consensus technique.  **Multivariate analysis** to adjust for potential confounders.  **Patient involvement**: The adapted version of the scale was assessed by a set of patient representatives. Participant recruitment done through patient organisation networks. |
| MacDonald 2015 (52);  Europe: UK;  Semi-structured interviews and consultation observation | Patients (18-65 y.o.), carers, stakeholders involved in cystic fibrosis care and research;  Cystic Fibrosis (CF) | 10 patients (NA); 2 carers (NA)^b38^ | Purposive sampling;  Multiple phases for qualitative analysis.  **Other validation activities**: Pilot observations and interviews (not disclosed if patients and/or carers took part in this pilot testing). |
| Boynton 2018 (53);  Outside Europe: USA;  Interviews | Patients (NA), carers, stakeholders involved in providing care for a cystic fibrosis diagnosis;  Cystic Fibrosis (CF) | 5 adult patients (NA), 2 parents of paediatric patients (NA) ^b39^ | Purposive sampling |
| Marinello 2019 (54);  Europe: countries not specified;  Focus groups | Patients (18-65 y.o.), clinicians;  Rare and Complex Connective Tissue and Musculoskeletal Diseases | NA (NA) ^b40^ | NA |
| Gutman 2018 (55);  Outside Europe: Australia, Canada, USA  Focus groups | Patients (18-65 y.o.; 65+ y.o.), carers;  Cystinosis; polycystic kidney disease; Focal segmental glomerulosclerosis; IgA nephropathy ^a41^ | 16 focus groups with 34 children (NA) and 62 parents (NA). | Purposive sampling;  Multiple phases for qualitative analysis;  Enhancing credibility of results (multiple researchers, checking with interviewees). |
| Grandjean 2021(56);  Europe: Switzerland;  Semi-structured interviews | Carers of patients (<18 y.o.);  Rare cancers | 31 families (46%): 12 mothers, 8 fathers, and 11 mother/father dyads. | Purposive sampling;  Multiple phases for qualitative analysis;  Enhancing credibility of results (multiple researchers, checking with interviewees). **Other validation activities:** Questions formulated based on Post-Intensive Care Syndrome in children (PICS-p Framework).  **Patient involvement:** Pilot interviews were conducted (carers piloted interview guides). |
| Hutchens 2022 (57);  Outside Europe: Australia;  Semi-structured interviews | Patients (18-65 y.o.);  Rare heart diseases: Arrhythmogenic right ventricular dysplasia, Long QT syndrome, Tetralogy of Fallot, Mitral valve prolapse (familial) | 25 (100%). | Purposive sampling;  Multiple phases for qualitative analysis;  Enhancing credibility of results (multiple researchers, checking with interviewees).  **Patient involvement**: Participant recruitment done though patient organisation networks. |
| de Dios Garcia-Diaz 2022 (58);  Europe: Spain;  Semi-structured interviews | Patients (<18 y.o.; 18-65 y.o.; 65+ y.o.), healthcare professionals involved in the care of lysosomal storage diseases;  Lysosomal Storage Diseases: Pompe Disease, Mucopolysaccharidosis, Gaucher Disease, Fabry Disease. | 20 patients (NA) ^b44^ | Purposive sampling;  Multiple phases for qualitative analysis;  Enhancing credibility of results (multiple researchers, checking with interviewees).  **Other validation activities:** Patient interviews.  **Patient involvement**: Patients were involved in priority setting (the main/most important needs/items). |
| Schmidt 2008 (59);  Europe: Austria, Germany, Greece, Netherlands, Sweden, UK;  Focus groups | Patients (<18 y.o.), carers, healthcare professionals;  Cystic fibrosis (CF) and juvenile rheumatoid arthritis ^a45^ | 204 parents (NA), 196 adolescent patients (NA)^b45^. | Purposive sampling.  **Other validation activities:** Focus groups with patients and parents to elicit items/statements, item prioritisation and reduction. |
| Feinberg 2013 (60);  Outside Europe: Canada;  Semi-structured interviews | Patients (18-65 y.o.; 65+ y.o.);  Neuroendocrine tumours (NET): small bowel, pancreas, gastric, bronchial, and unknown origin. | 18 (78%). | Purposive sampling;  Multiple phases for qualitative analysis;  Enhancing credibility of results (multiple researchers, checking with interviewees).  **Patient involvement:** Patients piloted interviews guides which included prompts to include topics not mentioned in the interview guide. |
| Simpson 2021 (61);  Europe: UK;  Semi-structured interviews | Patients (<18 y.o.; 18-65 y.o.), carers;  Several rare diseases (NA) | 7 patients (NA), 8 carers (NA). | Purposive sampling;  Enhancing credibility of results (multiple researchers, checking with interviewees)  **Patient involvement:** Participant recruitment done through patient organisation networks |
| Van den Steen 2022 (62);  Europe: Belgium;  Focus groups, semi-structured interviews | Patients (18-65 y.o.; 65+ y.o.);  Rare skin diseases: Bullous pemphigus, Granuloma annulare, Dermatomyositis, Pemphigus vulgaris, Systemic sclerosis (SS). | Interviews: 10 patients (NA). Focus groups: 2 patients (NA) ^b48^ | Purposive sampling;  Multiple phases for qualitative analysis;  Enhancing credibility of results (multiple researchers, checking with interviewees). **Other validation activities:** Study design was based on the Dutch guide for development of patient experience for measuring quality of care and included: 1) literature review, 2) semi-structured interviews, 3) focus groups and 4) mailing rounds with an expert panel that included patients.  **Patient involvement**: An expert panel that included patients reviewed the comprehensibility and validity of the drafted questions as well as participated in a prioritisation exercise. |
| Cuffe 2020 (63);  Europe: Ireland;  Unstructured interviews | Patients (18-65 y.o.; 65+ y.o.);  Multiple myeloma | 6 (NA) | Purposive sampling |
| Kuluski 2013 (64);  Outside Europe: Canada;  Semi-structured interviews | Patients (18-65 y.o.; 65+ y.o.);  Rare neurological diseases. | 110 (70%): 2 patients with rare neurological diseases. | Convenient sampling;  Enhancing credibility of results (multiple researchers, checking with interviewees).  **Other validation activities:** The design of the tool was based on a conceptual framework developed by the research team. The framework evolved from a scoping review of the literature and meetings with experts on the meaning of complexity in chronic disease. |
| **Mixed methods papers** | | | |
| Steinkamp 2010 (65);  Europe: Germany;  Focus groups, questionnaire | Patients (18-65 y.o.);  Cystic fibrosis (CF) | **Quantitative:** 389 participants (NA): adult patients (69%), parents (83%). **Qualitative**: 7 adult patients (NA), 13 parents (NA). | **Quantitative:** Existing validated scale(s): Picker Patient Experience Questionnaire(4);  Other validation activities: Focus groups (adult patients and carers) to design patient satisfaction items; interviews with professional experts.  **Patient involvement:** Patients and carers tested the survey in 4 centres.  **Qualitative:** NA. |
| Stahl 2015 (18);  Europe: Germany;  Focus groups, questionnaire | Patients (18-65 y.o.), carers, healthcare professionals;  Cystic fibrosis (CF) | **Quantitative**:  pilot study: 128 adult patients (70%), 108 parents (83%). Main study: 2,112 (74%). **Qualitative** **(focus groups)**: 20 patients and parents (NA) ^b52^ | **Quantitative:** Existing validated scale(s): generic items from the Picker Patient Experience Questionnaire (4);  Multivariate analysis to adjust for potential confounders;  Other validation activities: interviews with professionals; focus group with families; item-total correlation and Cronbach alpha coefficient (item internal consistency); exploratory factor analysis.  **Patient involvement**: Items reviewed by former focus group participants **Qualitative:** NA. |
| Spierings 2018 (66);  Europe: Netherlands  Questionnaire, interviews; focus groups | Patients (18-65 y.o.; 65+ y.o.);  Systemic sclerosis (SS) | **Quantitative**: 493 (24%). **Qualitative**: NA. | **Quantitative:** Existing validated scale(s): included items from the Consumer quality index (CQ index) for rheumatoid arthritis (11). **Qualitative:** Other validation activities:  focus groups and interviews. |
| Spierings 2019 (67);  Europe: Netherlands  Questionnaire, interviews; focus groups, working conference | Patients (18-65 y.o.); stakeholders involved in systemic sclerosis care and research;  Systemic sclerosis (SS) | **Quantitative**: 650 patients (31%) **Qualitative:** Focus groups and interviews: 23 patients (NA). Working conference: 10 patients (NA) | **Quantitative:** NA. **Qualitative:** Enhancing credibility of results (multiple researchers, checking with interviewees);  Other validation activities:  focus groups and interviews; working conference (nominal group technique) including patients in item refinement and selection exercises.  **Patient involvement**: Co-design of questionnaire with patients; patients were part of a pilot group discussion on the items; final pilot testing of the questionnaire by another group of patients. |
| Nevin 2021 (68);  Outside Europe: Australia  Questionnaire, semi-structured interviews | Carers of patients (18-65 y.o.);  Developmental and epileptic encephalopathies (DEEs) | 25 (93%) | **Quantitative:** Existing validated scale(s): none. **Qualitative:** Purposive sampling; Multiple phases for qualitative analysis; Enhancing credibility of results (multiple researchers, checking with interviewees); Other validation activities: In depth interviews with parents. |
| Al-Yateem 2016 (69);  Europe: Ireland  Questionnaire, semi-structured interviews | Patients (<18 y.o.; 18-65 y.o.);  Cystic fibrosis (CF) | **Quantitative:** 113 (NA). **Qualitative:** 25 (71%). | **Quantitative:** Other validation activities: questionnaire based on interviews on service quality; content validity process done by healthcare professionals working with CF adolescents and young adults. **Qualitative:** Purposive sampling; Interview script and technique piloted (although they do not disclose who participated in the pilot).  **Patient involvement:** Patients involved in item priority setting/rating exercise as well as reviewed the content adequacy and understandability of the questionnaire. |
| **Qualitative-quantitative papers** | | | |
| MacDonald 2017 (70);  Europe: UK;  Questionnaire, narrative questions | Patients (<18 y.o.; 18-65 y.o.);  Cystic fibrosis (CF) | **Quantitative:** NA. **Qualitative:** 7 (NA) | **Quantitative:** NA. **Qualitative:** Convenient sampling; Multiple phases for qualitative analysis. |
| Jarvis 2021 (71);  Europe: UK  Questionnaire, narrative questions | Patients (18-65 y.o.);  Inflammatory Bowel Disease (IBD), Cystic Fibrosis (CF) ^a58^ | 24 (22%) | NA |
| Palandri 2018 (72);  Europe: Italy  Questionnaire, narrative questions | Patients (18-65 y.o.), carers;  Myelofibrosis | 215 patients (75%), 62 carers (63%). | **Quantitative:** Existing validated scale(s): none. **Qualitative:** Multiple phases for qualitative analysis; Enhancing credibility of results (multiple researchers, checking with interviewees). |
| Van Groenendael 2015 (73);  Europe: UK  Questionnaire, narrative questions | Patients (18-65 y.o.), carers;  Alström syndrome | (23%) | **Quantitative:** NA.  **Qualitative:** Purposive sampling.  **Patient involvement:** Patients and carers were invited for pre-interview discussions, and this formed the basis of the structured interviews. |
| D'Aprano 2020 (74);  Outside Europe: Australia;  Questionnaire, narrative questions | Carers of patients (<18 y.o.; 18-65 y.o.);  Several are diseases: VACTERL, anorectal malformations, congenital limb malformations, Pierre Robin Sequence, Goldenhar syndrome, Astrocytoma, tracheo-oesophageal fistula, oesophageal atresia | 53 (51%) | **Quantitative:** NA.  **Qualitative:** Multiple phases for qualitative analysis. |

Legend:^a^ – papers including diseases other than rare and ultra-rare conditions; ^a21^ - platelet dysfunction ; ^a26^ – diabetes, multiple sclerosis and unspecified chronic skin, pulmonary and gastrointestinal conditions; ^a29, a45^ - asthma, diabetes, epilepsy, cerebral palsy, and atopic dermatitis; ^a34^ – chronic lymphoedema, encompassing secondary lymphoedema, lipoedema and lipolymphoedema; ^a41^ – encompasses more types of chronic kidney diseases, including congenital abnormalities of kidneys/urinary tract and Nephrotic syndrome (cause not specified) for which prevalence could not be assessed; ^a58^ - Diabetes, Nephrology (NA) and Haemoglobinopathies (NA); ^b^- papers including stakeholders other than patients and carers: ^b16^ – 64 health professionals involved in cystic fibrosis care; ^b19^ – 21 healthcare professionals; ^b38^ - 12 healthcare professionals observed but only 11 interviewed; ^b39^ - 3 physicians, 3 registered nurses, 9 respiratory therapists, 1 child life specialist (NA); ^b39^ – European Reference Network (ERN) ReCONNET clinicians (NA); ^b44^ - 25 healthcare professionals; ^b45^ – 32 healthcare professionals; ^b48^ - 10 other participants (including dermatologists and nurses);^;b52^ - 6 physicians, 6 nurses, 1 physiotherapist; ^b54^ – quantitative survey: 167 rheumatologists (48%), 51 specialised nurses (21%), 85 other health professionals (NA) / qualitative interviews: 12 rheumatologists, 5 specialised nurses / Working conference: 67 other participants, including 41 health professionals, 5 funders of scientific research and 15 members of the Arthritis Research and Collaboration Hub;

NA – Not applicable/available

**References**

1.	Husson O, Weidema M, Leonard H, Hartle DeYoung L, van der Graaf W, van de Poll‐Franse L. Supportive care needs of patients living with an extremely rare and unpredictable cancer: The Epithelioid Haemangioendothelioma patient experience. Eur J Cancer Care (Engl) [Internet]. 2021;30(6):1–11. Available from: https://search.ebscohost.com/login.aspx?direct=true&db=cin20&AN=153493799&site=ehost-live

2.	Boyes A, Girgis A, Lecathelinais C. Brief assessment of adult cancer patients’ perceived needs: development and validation of the 34-item Supportive Care Needs Survey (SCNS-SF34). J Eval Clin Pract [Internet]. 2009 Aug 1 [cited 2024 Jan 22];15(4):602–6. Available from: https://onlinelibrary.wiley.com/doi/full/10.1111/j.1365-2753.2008.01057.x

3.	Steinkamp G, Stahl K, Ellemunter H, Heuer E, S van KR, Busche M, et al. Cystic fibrosis (CF) care through the patients’ eyes - a nationwide survey on experience and satisfaction with services using a disease-specific questionnaire. Respir Med. 2015;109(1):79–87.

4.	Jenkinson C, Coulter A, Bruster S. The Picker Patient Experience Questionnaire: development and validation using data from in-patient surveys in five countries. International Journal for Quality in Health Care. 2002;14(5):353–8.

5.	Ellemunter H, Stahl K, Smrekar U, Steinkamp G. Evaluating patient experience in a cystic fibrosis centre using a disease-specific patient satisfaction questionnaire. Eur J Pediatr. 2015;174(11):1451–60.

6.	Britto MT, Slap GB, DeVellis RF, Hornung RW, Atherton HD, Knopf JM, et al. Specialists understanding of the health care preferences of chronically ill adolescents. J Adolesc Health. 2007;40(4):334–41.

7.	Cafaggi M, Marcucci M, Festini F, Braggion C. Expectations and needs of cystic fibrosis (CF) patients and the CF center’s response. Journal of Cystic Fibrosis. 2011;10(SUPPL. 1):S93-.

8.	Riekert KA, Eakin MN, Bilderback A, Ridge AK, Zhang J, Quittner AL. Adolescent and parent perceptions of their CF care team’s adherence promotion practices and satisfaction with care. Pediatr Pulmonol. 2012;47(SUPPL. 35):393–4.

9.	Heeres I. Partnership between parents of children with CF and the CF team at the Erasmus MC-sophia children’s hospital. Pediatr Pulmonol. 2015;50(SUPPL. 41):439.

10.	Spierings J, M van den ECH, Schriemer RM, J BMH, A van der BE, Bonte-Mineur F, et al. How do patients with systemic sclerosis experience currently provided healthcare and how should we measure its quality? Rheumatology (Oxford). 2020;59(6):1226–32.

11.	Zuidgeest M, Sixma H, Rademakers J. Measuring patients’ experiences with rheumatic care: The consumer quality index rheumatoid arthritis. Rheumatol Int [Internet]. 2009 Dec 16 [cited 2024 Jan 22];30(2):159–67. Available from: https://link.springer.com/article/10.1007/s00296-009-0926-3

12.	Berry L, Newson TP, Smith O. Benefits of patient feedback in quality improvement of a local cystic fibrosis service. Arch Dis Child. 2019;104(Supplement 2):A207–8.

13.	Riske B, Shearer R, Baker JR. Patient satisfaction with US Hemophilia Treatment Center Care, Teams and Services: The First National Survey. Haemophilia. 2020 Nov 1;26(6):991–8.

14.	Bramlett MD, Blumberg SJ, Ormson AE, George JM, Williams KL, Frasier AM, et al. Design and operation of the National Survey of Children with Special Health Care Needs, 2009-2010. Vital Health Stat . 2014;57(1):1–271.

15.	Agency for Healthcare Research and Quality. Consumer Assessment of Healthcare Providers and Systems (CAHPS) patient experience survey [Internet]. [cited 2024 Jan 4]. Available from: https://www.ahrq.gov/cahps/about-cahps/index.html

16.	Thomas A, Bradley J, Pickering N, Saba T. Patient experience of transition to a newly established cystic fibrosis service in the North West of England. Journal of Cystic Fibrosis. 2019;18(Supplement 1):S188-.

17.	Finlayson F. A New, Short Patient-Reported Experience Measure (Prem) for Cystic Fibrosis (CF) Care a- the Adult Cystic Fibrosis Experience (ACE) Measure. Respirology. 2020;25((Finlayson) Respiratory Medicine, Alfred, Melbourne, VIC, Australia):154.

18.	Stahl K, Steinkamp G, Ullrich G, Schulz W, S van KR, Heuer HE, et al. Patient experience in cystic fibrosis care: Development of a disease-specific questionnaire. Chronic Illn. 2015;11(2):108–25.

19.	Mitchell-Thain R, Yeoman A, Hegade V. Patient reported experiences through PBC foundation app: What impacts upon a PBC patient’s. experience? Gut. 2021;70(SUPPL 1):A39-.

20.	Sharif N, Shawcross A, Tahsin E, Davey S, Maitra A. Insights into parental experience of a specialist bronchiectasis and primary ciliary dyskinesia service provision-experience from a large tertiary paediatric centre in United Kingdom. Thorax. 2021;76(SUPPL 1):A157-.

21.	Hutchins K, Barr E, Bellcross C, Ali N, Guan Y, Hunt W. Evaluating potential differences in the disease experiences of adult minority patients with cystic. fibrosis. Journal of Cystic Fibrosis. 2021;20(Supplement 2):S141-.

22.	van Laar M, Glaser A, Phillips RS, Feltbower RG, Stark DP. The impact of a managed transition of care upon psychosocial characteristics and patient satisfaction in a cohort of adult survivors of childhood cancer. Psychooncology. 2013;22(9):2039–45.

23.	Absolom K, Greenfield D, Ross R, Horne B, Davies H, Glaser A, et al. Predictors of clinic satisfaction among adult survivors of childhood cancer. Eur J Cancer. 2006 Jul;42(10):1421–7.

24.	Pougheon Bertrand D, Minguet G, Gagnayre R, Lombrail P. Lessons from patient and parent involvement (P&PI) in a quality improvement program in cystic fibrosis care in France. Orphanet J Rare Dis. 2018;13(Suppl 1):19.

25.	Carman KL, Dardess P, Maurer M, Sofaer S, Adams K, Bechtel C, et al. Patient And Family Engagement: A Framework For Understanding The Elements And Developing Interventions And Policies Evidence & Potential. FEBRUARY. 2013;32(2).

26.	Homa K, Sabadosa KA, Marrow LC, Marshall BC. Experience of care from the perspective of individuals with cystic fibrosis and families: Results from 70 CF Foundation accredited programs in the USA. J Cyst Fibros. 2015;14(4):515–22.

27.	Homa K, Sabadosa KA, Nelson EC, Rogers WH, Marshall BC. Development and validation of a cystic fibrosis patient and family member experience of care survey. Qual Manag Health Care [Internet]. 2013 Apr [cited 2024 Jan 22];22(2):100–16. Available from: https://journals.lww.com/qmhcjournal/fulltext/2013/04000/development_and_validation_of_a_cystic_fibrosis.3.aspx

28.	Wong-Gibbons DL, Kancherla V, Romitti PA, Tyler MC, Damiano PC, Druschel CM, et al. Maternal reports of satisfaction with care and outcomes for children with craniosynostosis. J Craniofac Surg. 2009;20(1):138–42.

29.	Ananth P, Mun S, Reffat N, Kang SJ, Pitafi S, Ma X, et al. Refining Patient-Centered Measures of End-of-Life Care Quality for Children With Cancer. JCO Oncol Pract. 2022;18(3):e372–82.

30.	Baldelli I, Gallo F, Crimi M, Fregatti P, Mellini L, Santi P, et al. Experiences of patients with Poland syndrome of diagnosis and care in Italy: a pilot survey. Orphanet J Rare Dis. 2019;14(1):269.

31.	Hacker MR, Primeaux J, Manco-Johnson MJ. A patient satisfaction survey for haemophilia treatment centres. Haemophilia. 2006;12(2):163–8.

32.	Ware JE, Snyder MK, Wright WR, Davies AR. Defining and measuring patient satisfaction with medical care. Vol. 6, Evaluation andProgram Planning. 1983.

33.	MacKeigan LD, Larson LN. Development and validation of an instrument to measure patient satisfaction with pharmacy services. Med Care. 1989;27(5):552–536.

34.	Read N, Lim E, Tarzi MD, Hildick-Smith P, Burns S, Fidler KJ. Paediatric hereditary angioedema: a survey of UK service provision and patient experience. Clin Exp Immunol. 2014;178(3):483–8.

35.	Drewitz KP, Grey J, Brügmann P, Pichl J, Sammarco M, Aarts M, et al. Patients’ perception on the quality of care for multiple endocrine neoplasia disorders in Europe: an online survey from a patient support group. Endocrine. 2021;71(3):634–40.

36.	Rudebeck M, Scott C, Sireau N, Ranganath L. A patient survey on the impact of alkaptonuria symptoms as perceived by the patients and their experiences of receiving diagnosis and care. JIMD Rep. 2020;53(1):71–9.

37.	Schmidt S, Thyen U, Herrmann-Garitz C, Bomba F, Muehlan H. The Youth Health Care measure-satisfaction, utilization, and needs (YHC-SUN)-development of a self-report version of the Child Health Care (CHC-SUN) proxy-measure. BMC Health Serv Res. 2016;16:189.

38.	Schmidt S, Thyen U, Chaplin J, Mueller-Godeffroy E. Cross-cultural development of a child health care questionnaire on satisfaction, utilization, and needs. Ambul Pediatr. 2007;7(5):374–82.

39.	Chrysochoou EA, Hatziagorou E, Tsanakas J. Healthcare satisfaction, utilisation, and needs among families with cystic fibrosis. Journal of Cystic Fibrosis. 2019;18(Supplement 1):S174-.

40.	Thyen U, Ittermann T, Flessa S, Muehlan H, Birnbaum W, Rapp M, et al. Quality of health care in adolescents and adults with disorders/differences of sex development (DSD) in six European countries (dsd-LIFE). BMC Health Serv Res. 2018;18(1):527.

41.	Attkisson CC, Greenfield TK. The Client Satisfaction Questionnaire (CSQ) Scales [Internet]. 1996. Available from: https://www.researchgate.net/publication/265377848

42.	Sadlo A, Altevers J, Peplies J, Kaltz B, Claßen M, Bauer A, et al. Measuring satisfaction with health care in young persons with inflammatory bowel disease--an instrument development and validation study. BMC Health Serv Res. 2014;14:97.

43.	Spencer-Tansley R, Meade N, Ali F, Simpson A, Hunter A. Mental health care for rare disease in the UK - recommendations from a quantitative survey and multi-stakeholder workshop. BMC Health Serv Res. 2022;22(1):648.

44.	Lechevalier D, Sigg N, Humeau H, Vermersch C, Leducq S, Maruani A, et al. Healthcare transition from childhood to adulthood in pseudoxanthoma elasticum: Patient experience and recommendations for health practitioners. Ann Dermatol Venereol. 2022;

45.	Waldréus N, Jaarsma T, Ivarsson B, Strömberg A, Årestedt K, Kjellström B. Development and Validation of a Questionnaire to Measure Patient’s Experiences of Health Care in Pulmonary Arterial Hypertension Outpatient Clinics. Heart Lung Circ. 2019;28(7):1074–81.

46.	Herberger K, Blome C, Sandner A, Altheide F, Heyer K, Münter KC, et al. Quality of care of patients with chronic lymphoedema in Germany. Dermatology. 2013;226(3):238–46.

47.	Hytiris M, Johnston D, Mullen S, Smyth A, Dougan E, Rodie M, et al. Experience of health care at a reference centre as reported by patients and parents of children with rare conditions. Orphanet J Rare Dis. 2021;16(1):65.

48.	deJong NA, Wofford M, Song PH, Kappelman MD. Association of Care Coordination Experience and Health Services Use with Main Provider Type for Children with Inflammatory Bowel Disease. J Pediatr. 2021;234:142-148.e1.

49.	Gidengil C, Parast L, Burkhart ; Q, Brown J, Elliott MN, Lion ; K Casey, et al. Development and Implementation of the Family Experiences With Coordination of Care Survey Quality Measures [Internet]. Vol. 17, ACADEMIC PEDIATRICS. 2017. Available from: http://www.seattlechildrens.org/research/child-

50.	Guilabert M, Martínez-García A, Sala-González M, Solas O, Mira JJ. Results of a Patient Reported Experience Measure (PREM) to measure the rare disease patients and caregivers experience: a Spanish cross-sectional study. Orphanet J Rare Dis. 2021;16(1):67.

51.	Joaquín Mira J, Nuño-Solinís R, Guilabert-Mora M, Solas-Gaspar O, Fernández-Cano P, Asunción González-Mestre M, et al. Development and Validation of an Instrument for Assessing Patient Experience of Chronic Illness Care. Int J Integr Care. 2016;16(3):1–13.

52.	MacDonald K, Irvine L, Smith MC. An exploration of partnership through interactions between young “expert” patients with cystic fibrosis and healthcare professionals. J Clin Nurs. 2015;24(23–24):3528–37.

53.	Boynton BJ, Pike ML, Dean VL, Felten RB, Asiedu GB, Stellpflug CL, et al. Using qualitative analysis to improve cystic fibrosis care. Pediatr Pulmonol. 2018;53(Supplement 2):443.

54.	Marinello D, Ticciati S, Talarico R, Alexander T, Arnaud L, Cavagna L, et al. Identification of unmet needs related to rare and complex connective tissue and musculoskeletal diseases (rCTDs) across EU: The experience of the ern reconnet. Ann Rheum Dis. 2019;78(Supplement 2):999.

55.	Gutman T, Hanson CS, Bernays S, Craig JC, Sinha A, Dart A, et al. Child and Parental Perspectives on Communication and Decision Making in Pediatric CKD: A Focus Group Study. Am J Kidney Dis. 2018;72(4):547–59.

56.	Grandjean C, Ullmann P, Marston M, Maitre MC, Perez MH, Ramelet AS. Sources of Stress, Family Functioning, and Needs of Families With a Chronic Critically Ill Child: A Qualitative Study. Front Pediatr. 2021;9:740598.

57.	Hutchens J, Frawley J, Sullivan EA. The healthcare experiences of women with cardiac disease in pregnancy and postpartum: A qualitative study. Health Expect. 2022;

58.	de Dios García-Díaz J, López-Rodríguez M, Morales-Conejo M, Riera-Mestre A. Understanding the ecosystem of patients with lysosomal storage diseases in Spain: a qualitative research with patients and health care professionals. Orphanet J Rare Dis. 2022;17(1):17.

59.	Schmidt S, Thyen U, Chaplin J, Mueller-Godeffroy E, Bullinger M. Healthcare needs and healthcare satisfaction from the perspective of parents of children with chronic conditions: the DISABKIDS approach towards instrument development. Child Care Health Dev. 2008;34(3):355–66.

60.	Feinberg Y, Law C, Singh S, Wright FC. Patient experiences of having a neuroendocrine tumour: a qualitative study. Eur J Oncol Nurs. 2013;17(5):541–5.

61.	Simpson A, Bloom L, Fulop NJ, Hudson E, Leeson-Beevers K, Morris S, et al. How are patients with rare diseases and their carers in the UK impacted by the way care is coordinated? An exploratory qualitative interview study. Orphanet J Rare Dis. 2021;16(1):76.

62.	Van den Steen E, Ramaekers D, Horlait M, Gutermuth J. Development of a patient-reported experience measure for chronic inflammatory skin diseases. J Eur Acad Dermatol Venereol. 2022;36(6):913–21.

63.	Cuffe CH, Quirke MB, McCabe C. Patients’ experiences of living with multiple myeloma. Br J Nurs. 2020;29(2):103–10.

64.	Kuluski K, Hoang SN, Schaink AK, Alvaro C, Lyons RF, Tobias R, et al. The care delivery experience of hospitalized patients with complex chronic disease. Health Expect. 2013;16(4):e111-23.

65.	Steinkamp G, Stahl K, Ullrich G, Heuer E, Ellemunter H, Schwarz C, et al. CF care through the patient’s eyes - Development of a disease specific questionnaire measuring patient satisfaction with CF services. Journal of Cystic Fibrosis. 2010;9(SUPPL. 1):S100-.

66.	Spierings J, H VDEC, Schriemer MR, K DVBJ, Vonk MC. Patients’ evaluation of Dutch health care in systemic sclerosis: Unmet needs and preferences. Ann Rheum Dis. 2018;77(Supplement 2):521.

67.	Spierings J, C van den E, Schriemer R, L de P, Moens HB, J van L, et al. Optimal care for systemic sclerosis patients: recommendations from a patient-centered and multidisciplinary mixed-method study and working conference. Clin Rheumatol. 2019;38(4):1007–15.

68.	Nevin MS, Barlow-Stewart K, Wakefield EC, McGill CB, LeMarne F, Beavis E, et al. Uncovering the psychosocial impact of undergoing genetic testing for rare and recently discovered genetic epilepsies. Twin Research and Human Genetics. 2021;24(1):71.

69.	Al-Yateem N, Docherty C, Rossiter R. Determinants of Quality of Care for Adolescents and Young Adults With Chronic Illnesses: A Mixed Methods Study. J Pediatr Nurs. 2016;31(3):255–66.

70.	MacDonald K, Mathews A. Exploring experiences of the in-patient ward round with young people with CF. Journal of Cystic Fibrosis. 2017;16(Supplement 1):S163-.

71.	Jarvis C, Thompson A, Marshall L, Fletcher N, Minson S. Patient experiences of transition from paediatric to adult services. Arch Dis Child. 2021;106(SUPPL 1):A240-.

72.	Palandri F, Benevolo G, Iurlo A, Abruzzese E, Carella AM, Paoli C, et al. Life for patients with myelofibrosis: the physical, emotional and financial impact, collected using narrative medicine-Results from the Italian “Back to Life” project. Qual Life Res. 2018;27(6):1545–54.

73.	Van Groenendael S, Giacovazzi L, Davison F, Holtkemper O, Huang Z, Wang Q, et al. High quality, patient centred and coordinated care for Alstrom syndrome: a model of care for an ultra-rare disease. Orphanet J Rare Dis. 2015;10:149.

74.	D’Aprano A, Gibb S, Riess S, Cooper M, Mountford N, Meehan E. Important components of a programme for children with medical complexity: An Australian perspective. Child Care Health Dev. 2020;46(1):90–103.
