# Supplementary material for: Patient-reported experiences of people living with a rare disease with secondary and tertiary care: a scoping review
Source: Orphanet J Rare Dis. 2026 Nov 4;21:388. doi: 10.1186/s13023-026-04368-5 (PMC13733059; doi:10.1186/s13023-026-04368-5)
Supplement: Supplementary file 4 [file 13023_2026_4368_MOESM4_ESM.docx]

**Additional file 4**. Mapping of questionnaires against the domains and categories of healthcare experience for people living with a rare disease (PLWRD) of secondary and tertiary healthcare settings.

| **Questionnaire**  **Reference (No)** | **Rare disease specific** | **Domains**  Number and name | **Categories**  Number and name | | | **Full questionnaire available / accessible** |
| --- | --- | --- | --- | --- | --- | --- |
| **Generic instruments (n= 13)** |  |  |  | | |  |
| **Fully validated instruments (n= 7)** | | | | | | |
| **Q1. Youth Healthcare measure-satisfaction, utilization, and needs (YHC-SUN)**  Developed and validated by: (4)  Applied by: (6) | Not exclusively but tested and validated in over 30 rare diseases, including CF | **Total = 9/11** | | | **Total = 30/71** | Yes |
|  |  | 1.Access | | | 1.1. Duration of appointments (little time/enough time); 1.2. Waiting time (in the waiting room, to get an appointment after doing tests, outside of routine visits, unexpected cancellations); 1.5. Mode of appointment (in person, phone, video call) – acceptability. |  |
|  |  | 2. Comprehensiveness | | | 2.1. Access to multidisciplinary care; 2.2. Psychological/emotional support (patient and/or carer); 2.3. Access to specialized providers; 2.4. Peer-support (patient organisation or other): existing peer-support, access to peer-support; 2.5. Involvement of/access to social worker; 2.6. Guidance about social services and education resources; 2.9. Liaison and collaboration with schools and school-related services (awareness, liaison with school nurses, learning interventions, systematic instructions in school by HCPs); 2.11. Supply/access to medical/physical aid equipment, home care; 2.12. Respite care and bereavement support (for family carers) |  |
|  |  | 3.Continuity | | | 3.1. Access to and satisfaction with emergency care. |  |
|  |  | 4.Coordination | | | 4.2. Effectiveness of how care is coordinated. |  |
|  |  | 5.People-centredness | | | 5.1. HCP communication / listening skills; 5.2. Patient/carer participation/involvement in care decision-making; 5.3. Preferences of patient taken into account / involved in decision-making; 5.4. HCP respect, empathy, and friendliness. |  |
|  |  | 7.Self-management support | | | 7.1. Information provided on the rare condition (before /during / after diagnosis); 7.2. Information on treatment (clear info on objectives, results...); 7.4. Peer-support (patient organisation or other): informed of availability, given contact; 7.7. 7.7. Education/self-management programmes |  |
|  |  | 9.Overall perceived quality of care | | | 9.1. Technical/Instrumental quality of care: Perceived skills of the care team (including knowledge levels / expertise of the healthcare professionals providing care); 9.2. Overall satisfaction with care; 9.3. Overall experience with the care received |  |
|  |  | 10.Diagnosis & treatment/interventions | | | 10.1. Diagnosis (timely, misdiagnosis, length of diagnosis journey, emotional impact, feeling dismissed); 10.2. Access to treatment (medication refills, emergency medication, inpatient treatment, home treatment, local hospital, bureaucracy); 10.3. Treatment effectiveness and satisfaction (existence and/or access to guidelines or other validated tools to assess and monitor response); 10.5. Adherence to treatment (e.g., discussing/addressing barriers to adherence). |  |
|  |  | 11.Service aspect of care | | | 11.1. Premises/ Facilities (cleanliness, toilets, seating, room design and furnishing, functionality of care spaces, play areas, availability of food/snacks). |  |
| **Q2. Child Health Care Questionnaire - Satisfaction, Utilization and Needs (CHC-SUN)**  Developed and validated by: (1,2)  Applied by:(3)  Adapted by: (4) | Not exclusively but tested and validated in cystic fibrosis (CF) | **Total = 9/11** | | | **Total = 25/71** | Partially |
|  |  | 1.Access | | | 1.4. Location of appointments - convenience; 1.5. Mode of appointment (in person, phone, video call) – acceptability; 1.6. Insurance coverage and financial burden (parking, travelling, testing costs, access other types of financial support and reimbursement of care) |  |
|  |  | 2. Comprehensiveness | | | 2.2. Psychological/emotional support (patient and/or carer); 2.3. Access to specialized providers; 2.4. Peer-support (patient organisation or other): existing peer-support, access to peer-support; 2.5. Involvement of/access to social worker; 2.6. Guidance about social services and education resources; 2.7. Guidance on procedures for disability status and fiscal benefits; 2.9. Liaison and collaboration with schools and school-related services (awareness, liaison with school nurses, learning interventions, systematic instructions in school by HCPs); 2.11. Supply/access to medical/physical aid equipment, home care; 2.12. Respite care and bereavement support (for family carers). |  |
|  |  | 3.Continuity | | | 3.4. Access to and satisfaction with emergency care. |  |
|  |  | 4.Coordination | | | 4.2. Effectiveness of how care is coordinated. |  |
|  |  | 5.People-centredness | | | 5.2. Patient/carer participation/involvement in care decision-making; 5.3. Preferences of patient taken into account / involved in decision-making; 5.4. HCP respect, empathy and friendliness. |  |
|  |  | 7.Self-management support | | | 7.1. Information provided on the rare condition (before /during / after diagnosis); 7.2. Information on treatment (clear info on objectives, results...); 7.4. Peer-support (patient organisation or other): informed of availability, given contact; 7.7. 7.7. Education/self-management programmes |  |
|  |  | 9.Overall perceived quality of care | | | 9.2. Overall satisfaction with care. |  |
|  |  | 10.Diagnosis & treatment/interventions | | | 10.2. Access to treatment (medication refills, emergency medication, inpatient treatment, home treatment, local hospital, bureaucracy); 10.5. Adherence to treatment (e.g. discussing/addressing barriers to adherence). |  |
|  |  | 11.Service aspect of care | | | 11.1. Premises/ Facilities (cleanliness, toilets, seating, room design and furnishing, functionality of care spaces, play areas, availability of food/snacks). |  |
| **Q3. Health Care Preferences of Chronically Ill Adolescents**  Developed and validated by: (11)  Applied by: (12) | Not exclusively but validated for CF, inflammatory bowel disease, juvenile rheumatoid arthritis | **Total = 9/11** | | | **Total = 16/71** | Yes |
|  |  | 1.Access | | | 1.1. Duration of appointments (little time/enough time); 1.4. Location of appointments – convenience. |  |
|  |  | 2.Comprehensiveness | | | 2.10. Healthcare professionals asked about the impact of the disease on patient’s / carer’s life; 2.14. Questions on the patient’s health habits. |  |
|  |  | 3.Continuity | | | 3.1. Is there a named healthcare professional who they can contact / how contactable are they. |  |
|  |  | 5.People-centredness | | | 5.1. HCP communication / listening skills; 5.2. Patient/carer participation/involvement in care decision-making; 5.3. Preferences of patient taken into account / involved in decision-making; 5.4. HCP respect, empathy and friendliness. |  |
|  |  | 6.Safety | | | 6.2. Infection prevention measures, e.g., hand hygiene. |  |
|  |  | 7.Self-management support | | | 7.3. Quality of the information provided (accessibility, effectiveness / accuracy, timeliness, understandability, format); 7.6. Information on tests and test results. |  |
|  |  | 8.Trust | | | 8.1. Trust / confidence in the care team |  |
|  |  | 9.Overall perceived quality of care | | | 9.1. Technical/Instrumental quality of care: Perceived skills of the care team (including knowledge levels / expertise of the healthcare professionals providing care). |  |
|  |  | 11.Service aspect of care | | | 11.1. Premises/ Facilities (cleanliness, toilets, seating, room design and furnishing, functionality of care spaces, play areas, availability of food/snacks); 11.2. Friendliness of support staff. |  |
| **Q4. Genetic Counselling Satisfaction Scale (GCSS)**  Developed and validated by: (9)  Applied by: (10) | Not exclusively | **Total = 5/11** | | | **Total = 5/71** | Yes |
|  |  | 1.Access | | | 1.1. Duration of appointments (little time/enough time). |  |
|  |  | 2.Comprehensiveness | | | 2.10. Healthcare professionals asked about the impact of the disease on patient’s / carer’s life. |  |
|  |  | 5.People-centredness | | | 5.4. HCP respect, empathy and friendliness. |  |
|  |  | 7.Self-management support | | | 7.3. Quality of the information provided (accessibility, effectiveness / accuracy, timeliness, understandability, format). |  |
|  |  | 9.Overall quality of care | | | 9.2. Overall satisfaction with care. |  |
| **Q5. Supportive Care Needs Survey Short Form (SCNS-SF34)**  Developed and validated by: (13)  Applied by: (14) | No | **Total = 4/11** | | | **Total = 10/71** | Yes |
|  |  | 2.Comprehensiveness | | | 2.1. Access to multidisciplinary care; 2.2. Psychological/emotional support (patient and/or carer); 2.10. Healthcare professionals asked about the impact of the disease on patient’s / carer’s life. |  |
|  |  | 3.Continuity | | | 3.1. Is there a named healthcare professional who they can contact / how contactable are they. |  |
|  |  | 5.People-centredness | | | 5.1. HCP communication / listening skills; 5.2. Patient/carer participation/involvement in care decision-making; 5.3. Preferences of patient taken into account / involved in decision-making. |  |
|  |  | 7.Self-management support | | | 7.2. Information on treatment (clear info on objectives, results...); 7.6. Information on tests and test results; 7.8. Information on everyday management and daily life (sex life, sports). |  |
| **Q6. Family Experiences with Coordination of Care Survey**  Developed and validated by: (7)  Applied by: (8) | No | **Total = 5/11** | | | **Total = 17/71** | Yes |
|  |  | 2.Comprehensiveness | | | 2.1. Access to multi-disciplinary care; 2.3. Access to specialized providers; 2.6. Guidance about social services and education resources; 2.9. Liaison and collaboration with schools and school-related services (awareness, liaison with school nurses, learning interventions, systematic instructions in school by HCPs); 2.10. Healthcare professionals asked about the impact of the disease on patient’s / carer’s life. |  |
|  |  | 3.Continuity | | | 3.4. Access to and satisfaction with emergency care; 3.5. Access to and satisfaction with transition of paediatric to adult care. |  |
|  |  | 4.Coordination | | | 4.3. Given a care plan (written / digital); 4.5. Existence of a case manager / care coordinator; 4.6. Access to medical records/ history/ registries. |  |
|  |  | 5.People-centredness | | | 5.1. HCP communication / listening skills; 5.2. Patient/carer participation/involvement in care decision-making |  |
|  |  | 7.Self-management support | | | 7.3. Quality of the information provided (accessibility, effectiveness / accuracy, timeliness, understandability, format); 7.9. Information on elective admissions and upon hospital discharge. |  |
| **Q7. Customer satisfaction questionnaire (CSQ-4)**  Developed and validated by: (5)  Applied by: (6) | No | **Total =1/11** | | | **Total = 2/71** | Yes |
|  |  | 9.Overall perceived quality of care | | | 9.2. Overall satisfaction with care; 9.3. Overall experience with the care received |  |
| **Partially validated and/or unvalidated author-built questionnaires (n=6)** | | | | | | |
| **Q8. Patient experiences of transition from paediatric to adult services**  Developed and applied by: (27) | No, not exclusively but tested in Inflammatory Bowel Disease, and CF | **Total = 5/11** | | | **Total = 10/71** | No |
|  |  | 1.Access | | | 1.2. Waiting time (in the waiting room, to get an appointment after doing tests, outside of routine visits, unexpected cancellations); 1.4. Timing of appointments – suitability. |  |
|  |  | 2.Comprehensiveness | | | 2.13. Care adapted to the evolving patient needs (related to age, independent living, starting a relationship, starting a job). |  |
|  |  | 3.Continuity | | | 3.3. Care continuity; 3.5. Access to and satisfaction with transition of paediatric to adult care. |  |
|  |  | 5.People-centredness | | | 5.1. HCP communication / listening skills; 5.2. Patient/carer participation/involvement in care decision-making; 5.3. Preferences of patient taken into account / involved in decision-making. |  |
|  |  | 7.Self-management support | | | 7.1. Information provided on the rare condition (before /during / after diagnosis); 7.3. Quality of the information provided (accessibility, effectiveness / accuracy, timeliness, understandability, format). |  |
| **Q9. Determinants of Quality of Care for Adolescents and Young Adults With Chronic Illnesses**  Developed and applied by: (24) | Yes; CF | **Total = 5/11** | | | **Total = 15/71** | Partially |
|  |  | 2.Comprehensiveness | | | 2.10. Healthcare professionals asked about the impact of the disease on patient’s / carer’s life; 2.13. Care adapted to the evolving patient needs (related to age, independent living, starting a relationship, starting a job); 2.14. Questions on the patient’s health habits. |  |
|  |  | 4.Coordination | | | 4.3. Given a care plan (written / digital). |  |
|  |  | 5.People-centredness | | | 5.1. HCP communication / listening skills; 5.2. Patient/carer participation/involvement in care decision-making; 5.3. Preferences of patient taken into account / involved in decision-making. |  |
|  |  | 7.Self-management support | | | 7.1. Information provided on the rare condition (before /during / after diagnosis); 7.2. Information on treatment (clear info on objectives, results...); 7.3. Quality of the information provided (accessibility, effectiveness / accuracy, timeliness, understandability, format); Information for carers and family members; 7.5. Information on where to turn with questions (person / website / support group); 7.7. Education/self-management programmes; 7.8. Information on everyday management and daily life (sex life, sports); |  |
|  |  | 9.Overall perceived quality of care | | | 9.1. Technical/Instrumental quality of care: Perceived skills of the care team (including knowledge levels / expertise of the healthcare professionals providing care). |  |
| **Q10. IEXPAC-rare-diseases**  Developed and applied by: (26) | Yes | **Total = 7/11** | | | **Total = 21/71** | Yes |
|  |  | 1.Access | | | 1.4. Location of appointments - convenience; 1.7. How the appointment was made (in person, by phone, online…) – easiness. |  |
|  |  | 2. Comprehensiveness | | | 2.3. Access to specialized providers; 2.6. Guidance about social services and education resources; 2.10. Healthcare professionals asked about the impact of the disease on patient’s / carer’s life; 2.14. Questions on the patient’s health habits |  |
|  |  | 3.Continuity | | | 3.1. Is there a named healthcare professional who they can contact / how contactable are they; 3.2. Follow-up path planned after the visit; 3.4. Access to and satisfaction with emergency care. |  |
|  |  | 4.Coordination | | | 4.1. Team working of the healthcare professionals involved in care (including case conferences); 4.2. Effectiveness of how care is coordinated; 4.3. Given a care plan (written / digital); Access medical records/ history/registries; 4.4. Transfer of information to the local doctor: specialist / primary care central / GP (between hospitals/clinics). |  |
|  |  | 5.People-centredness | | | 5.1. HCP communication / listening skills; 5.3. Preferences of patient taken into account / involved in decision-making; 5.4. HCP respect, empathy and friendliness. |  |
|  |  | 7.Self-management support | | | 7.1. Information provided on the rare condition (before /during / after diagnosis); 7.2. Information on treatment (clear info on objectives, results...); 7.4. Peer-support (patient organisation or other): informed of availability, given contact; 7.5. Information on where to turn with questions (person / website / support group); 7.6. Information on tests and test results; |  |
|  |  | 10.Diagnosis & treatment/interventions | | | 10.5. Adherence to treatment (e.g. discussing/addressing barriers to adherence). |  |
| **Q11. Rare disease patient experiences of health service support and mental health**  Developed and applied by: (28) | Yes | **Total = 7/11** | | | **Total = 15/71** | Partially |
|  |  | 1.Access | | | 1.2. Waiting time (in the waiting room, to get an appointment after doing tests, outside of routine visits, unexpected cancellations); 1.4. Location of appointments – convenience. |  |
|  |  | 2. Comprehensiveness | | | 2.1. Access to multidisciplinary care; 2.2. Psychological/emotional support (patient and/or carer); 2.5. Involvement of/access to social worker; 2.7. Guidance on procedures for disability status and fiscal benefits; 2.12. Respite care and bereavement support (for family carers). |  |
|  |  | 4.Coordination | | | 4.2. Effectiveness of how care is coordinated. |  |
|  |  | 5.People-centredness | | | 5.1. HCP communication / listening skills; 5.4. HCP respect, empathy and friendliness. |  |
|  |  | 7.Self-management support | | | 7.1. Information provided on the rare condition (before /during / after diagnosis); 7.3. Quality of the information provided (accessibility, effectiveness / accuracy, timeliness, understandability, format); 7.4. Peer-support (patient organisation or other): informed of availability, given contact. |  |
|  |  | 9.Overall perceived quality of care | | | 9.1. Technical/Instrumental quality of care: Perceived skills of the care team (including knowledge levels / expertise of the healthcare professionals providing care) |  |
|  |  | 10.Diagnosis & treatment/interventions | | | 10.1. Diagnosis (timely, misdiagnosis, length of diagnosis journey, emotional impact, feeling dismissed); 10.2. Access to treatment (medication refills, emergency medication, inpatient treatment, home treatment, local hospital, bureaucracy); 10.3. Treatment effectiveness and satisfaction (existence and/or access to guidelines or other validated tools to assess and monitor response). |  |
| **Q12. Value Of The Complex Care Service**  Developed and applied by: (29) | Yes | **Total = 7/11** | | | **Total = 13/71** | Yes |
|  |  | 1.Access | | | 1.2. Waiting time (in the waiting room, to get an appointment after doing tests, outside of routine visits, unexpected cancellations); 1.4. Timing of appointments - suitability; 1.5. Mode of appointment (in person, phone, video call) – acceptability. |  |
|  |  | 2.Comprehensiveness | | | 2.6. Guidance about social services and education resources. |  |
|  |  | 3.Continuity | | | 3.1. Is there a named healthcare professional who they can contact / how contactable are they. |  |
|  |  | 4.Coordination | | | 4.2. Effectiveness of how care is coordinated; 4.3. Given a care plan (written / digital); 4.5. Existence of a case manager / care coordinator; 4.6. Access to medical records/ history/ registries. |  |
|  |  | 5.People-centredness | | | 5.4. HCP respect, empathy and friendliness. |  |
|  |  | 7.Self-management support | | | 7.3. Quality of the information provided (accessibility, effectiveness / accuracy, timeliness, understandability, format). |  |
|  |  | 9.Overall perceived quality of care | | | 9.2. Overall satisfaction with care; 9.3. Overall experience with the care received |  |
| **Q13. Experience of healthcare at reference centres of patients and parents of children with rare conditions**  Developed and applied by: (25) | Yes | **Total = 6/11** | | | **Total = 14/71** | Yes |
|  |  | 2.Comprehensiveness | | | 2.1. Access to multidisciplinary care; 2.2. Psychological/emotional support (patient and/or carer); 2.4. Peer-support (patient organisation or other): existing peer-support, access to peer-support |  |
|  |  | 3.Continuity | | | 3.1. Is there a named healthcare professional who they can contact / how contactable are they. |  |
|  |  | 4.Coordination | | | 4.1. Team working of the healthcare professionals involved in care (including case conferences); 4.2. Effectiveness of how care is coordinated. |  |
|  |  | 5.People-centredness | | | 5.2. Patient/carer participation/involvement in care decision-making. |  |
|  |  | 7.Self-management support | | | 7.1. Information provided on the rare condition (before /during / after diagnosis); 7.3. Quality of the information provided (accessibility, effectiveness / accuracy, timeliness, understandability, format); 7.4. Peer-support (patient organisation or other): informed of availability, given contact. |  |
|  |  | 9. Overall perceived quality of care | | | 9.1. Technical/Instrumental quality of care: Perceived skills of the care team (including knowledge levels / expertise of the healthcare professionals providing care); 9.2. Overall satisfaction with care. 9.4. Patient participation in research. |  |
|  |  | 10.Diagnosis & treatment/interventions | | | 10.1. Diagnosis (timely, misdiagnosis, length of diagnosis journey, emotional impact, feeling dismissed). |  |
| **Disease-specific instruments (n=30)** | | | | | | |
| **Fully validated instruments (n= 5)** | | | | | | |
| **Q14. CF patient experience and satisfaction with care services**  Developed, validated and applied by: (12,17–19) | Yes; CF | **Total = 11/11** | | | **Total = 31/71** | Yes |
|  |  | 1.Access | | | 1.1. Duration of appointments (little time/enough time); 1.2. Waiting time (in the waiting room, to get an appointment after doing tests, outside of routine visits, unexpected cancellations); 1.3. Timing of appointments – suitability. |  |
|  |  | 2. Comprehensiveness | | | 2.1. Access to multidisciplinary care; 2.2. Psychological/emotional support (patient and/or carer); 2.5. Involvement of/access to social worker; 2.13. Care adapted to the evolving patient needs (related to age, independent living, starting a relationship, starting a job). |  |
|  |  | 3.Continuity | | | 3.1. Is there a named healthcare professional who they can contact / how contactable are they; 3.2. Follow-up path planned after the visit/ hospital discharge (including booking of regular examinations...); 3.3. Care continuity; 3.4. Access to and satisfaction with emergency care. |  |
|  |  | 4.Coordination | | | 4.1. Team working of the healthcare professionals involved in care (including case conferences). |  |
|  |  | 5.People-centredness | | | 5.1. HCP communication / listening skills; 5.2. Patient/carer participation/involvement in care decision-making; 5.3. Preferences of patient taken into account / involved in decision-making; 5.4. HCP respect, empathy and friendliness. |  |
|  |  | 6.Safety | | | 6.1. Safety of care and of care spaces (including patient segregation if/when necessary). |  |
|  |  | 7.Self-management support | | | 7.1. Information provided on the rare condition (before /during / after diagnosis); 7.2. Information on treatment (clear info on objectives, results...); 7.6. Information on tests and test results; 7.8. Information on everyday management and daily life (sex life, sports); Information for carers and family members; 7.9. Information on elective admissions and upon hospital discharge; 7.10. Info on research and registries. |  |
|  |  | 8.Trust | | | 8.1. Trust / confidence in the care team. |  |
|  |  | 9.Overall perceived quality of care | | | 9.1. Technical/Instrumental quality of care: Perceived skills of the care team (including knowledge levels / expertise of the healthcare professionals providing care). |  |
|  |  | 10.Diagnosis & treatment/interventions | | | 10.2. Access to treatment (medication refills, emergency medication, inpatient treatment, home treatment, local hospital, bureaucracy). |  |
|  |  | 11.Service aspects of care | | | 11.1. Premises/ Facilities (cleanliness, toilets, seating, room design and furnishing, functionality of care spaces, play areas, availability of food/snacks); 11.6. Accommodation for close family; 11.2. Friendliness of support staff; Toys provided |  |
| **Q15. CF patient and family experience of care survey**  Developed and validated by: (20)  Applied by: (21) | Yes; CF | **Total = 7/11** | | | **Total = 16/71** | Partially |
|  |  | 1.Access | | | 1.1. Duration of appointments (little time/enough time); 1.2. Waiting time (in the waiting room, to get an appointment after doing tests, outside of routine visits, unexpected cancellations); 1.4. Timing of appointments – suitability. |  |
|  |  | 2. Comprehensiveness | | | 2.1. Access to multidisciplinary care; 2.2. Psychological/emotional support (patient and/or carer); 2.5. Involvement of/access to social worker. |  |
|  |  | 4.Coordination | | | 4.1. Team working of the healthcare professionals involved in care (including case conferences). |  |
|  |  | 5.People-centredness | | | 5.1. HCP communication / listening skills; 5.2. Patient/carer participation/involvement in care decision-making; 5.4. HCP respect, empathy and friendliness. |  |
|  |  | 7.Self-management support | | | 7.2. Information on treatment (clear info on objectives, results...). |  |
|  |  | 9.Overall perceived quality of care | | | 9.1. Technical/Instrumental quality of care: Perceived skills of the care team (including knowledge levels / expertise of the healthcare professionals providing care); 9.2. Overall satisfaction with care; 9.3. Overall experience with the care received. |  |
|  |  | 10.Diagnosis & treatment/interventions | | | 10.2. Access to treatment (medication refills, emergency medication, inpatient treatment, home treatment, local hospital, bureaucracy); 10.3. Treatment effectiveness and satisfaction (existence and/or access to guidelines or other validated tools to assess and monitor response) |  |
| **Q16. Clinic satisfaction among adult survivors of childhood cancer* (2 questionnaire administered at different timepoints)**  Developed and validated by: (15)  Applied by: (16) | Yes; paediatric cancer | **Total = 7/11** | | | **Total = 17/71** | Partially |
|  |  | 1.Access | | | 1.1. Duration of appointments (little time/enough time); 1.2. Waiting time (in the waiting room, to get an appointment after doing tests, outside of routine visits, unexpected cancellations); 1.5. Mode of appointment (in person, phone, video call) – acceptability. |  |
|  |  | 2.Comprehensiveness | | | 2.1. Access to multi-disciplinary care; 2.2. Psychological/emotional support (patient and/or carer); 2.3. Access to specialized providers; 2.13. Care adapted to the evolving patient needs (related to age, independent living, starting a relationship, starting a job). |  |
|  |  | 3.Continuity | | | 3.2. Follow-up path planned after the visit/ hospital discharge (including booking of regular examinations...). |  |
|  |  | 4.Coordination | | | 4.4. Transfer of information to the local doctor: specialist / primary care central / GP (between hospitals/clinics). |  |
|  |  | 5.People-centredness | | | 5.1. HCP communication / listening skills; 5.4. HCP respect, empathy and friendliness. |  |
|  |  | 7.Self-management support | | | 7.2. Information on treatment (clear info on objectives, results...); 7.8. Information on everyday management and daily life (sex life, sports); 7.13. Information on reimbursement of care. |  |
|  |  | 9.Overall quality of care | | | 9.1. Technical/Instrumental quality of care: Perceived skills of the care team (including knowledge levels / expertise of the healthcare professionals providing care); 9.2. Overall satisfaction with care; 9.3. Overall experience with the care received. |  |
| **Q17. Patient’s Experiences of Health Care in Pulmonary Arterial Hypertension Outpatient Clinics (PAHC-PREM)**  Developed, validated and applied by: (22) | Yes; Pulmonary Arterial Hypertension | **Total = 5/11** | | | **Total = 9/71** | Yes |
|  |  | 1.Access | | | 1.2. Waiting time (in the waiting room, to get an appointment after doing tests, outside of routine visits, unexpected cancellations); 1.4. Timing of appointments – suitability. |  |
|  |  | 2.Comprehensiveness | | | 2.10. Healthcare professionals asked about the impact of the disease on patient’s / carer’s life. |  |
|  |  | 4.Coordination | | | 4.2. Effectiveness of how care is coordinated. |  |
|  |  | 5. People-centredness | | | 5.1. HCP communication / listening skills; 5.2. Patient/carer participation/involvement in care decision-making. |  |
|  |  | 7.Self-management support | | | 7.1. Information provided on the rare condition (before /during / after diagnosis); 7.2. Information on treatment (clear info on objectives, results...); 7.8. Information on everyday management and daily life (sex life, sports). |  |
| **Q18. Satisfaction with care in young persons with Inflammatory bowel disease (IBD)**  Developed, validated and applied by: (23) | Patients; IBD | **Total = 9/11** | | | **Total = 25/71** | Yes |
|  |  | 1.Access | | | 1.1. Duration of appointments (little time/enough time); 1.2. Waiting time (in the waiting room, to get an appointment after doing tests, outside of routine visits, unexpected cancellations); 1.4. Timing of appointments - suitability; 1.6. Insurance coverage and financial burden (parking, travelling, testing costs, access other types of financial support and reimbursement of care). |  |
|  |  | 2. Comprehensiveness | | | 2.1. Access to multidisciplinary care; 2.2. Psychological/emotional support (patient and/or carer); 2.3. Access to specialized providers; 2.4. Peer-support (patient organisation or other): existing peer-support, access to peer-support; 2.10. Healthcare professionals asked about the impact of the disease on patient’s / carer’s life; 2.13. Care adapted to the evolving patient needs (related to age, independent living, starting a relationship, starting a job) |  |
|  |  | 3.Continuity | | | 3.1. Is there a named healthcare professional who they can contact / how contactable are they; 3.4. Access to and satisfaction with emergency care. |  |
|  |  | 4.Coordination | | | 4.1. Team working of the healthcare professionals involved in care (including case conferences); 4.4. Transfer of information to the local doctor: specialist / primary care central / GP (between hospitals/clinics). |  |
|  |  | 5.People-centredness | | | 5.1. HCP communication / listening skills; 5.2. Patient/carer participation/involvement in care decision-making; 5.3. Preferences of patient taken into account / involved in decision-making; 5.4. HCP respect, empathy and friendliness. |  |
|  |  | 7.Self-management support | | | 7.3. Quality of the information provided (accessibility, effectiveness / accuracy, timeliness, understandability, format); 7.4. Peer-support (patient organisation or other): informed of availability, given contact; 7.6. Information on tests and test results. |  |
|  |  | 9.Overall perceived quality of care | | | 9.1. Technical/Instrumental quality of care: Perceived skills of the care team (including knowledge levels / expertise of the healthcare professionals providing care). |  |
|  |  | 10.Diagnosis & treatment/interventions | | | 10.2. Access to treatment (medication refills, emergency medication, inpatient treatment, home treatment, local hospital, bureaucracy). |  |
|  |  | 11.Service aspect of care | | | 11.1. Premises/ Facilities (cleanliness, toilets, seating, room design and furnishing, functionality of care spaces, play areas, availability of food/snacks); 11.2. Friendliness of support staff. |  |
| **Partially validated and/or unvalidated author-built questionnaires (n=24)** | | | | | | |
| **Q19. Adult Cystic fibrosis Experience (ACE) Score**  Developed and applied by: (30) | Yes; CF | **Total = 8/11** | | | **Total = 11/71** | Partially |
|  |  | 1.Access | | | 1.2. Waiting time (in the waiting room, to get an appointment after doing tests, outside of routine visits, unexpected cancellations); 1.4. Timing of appointments – suitability. |  |
|  |  | 2.Comprehensiveness | | | 2.1. Access to multidisciplinary care; 2.3. Access to specialised providers. |  |
|  |  | 3.Continuity | | | 3.1. Is there a named healthcare professional who they can contact / how contactable are they. |  |
|  |  | 4.Coordination | | | 4.7. All specialists needed to address the case were present during consultations. |  |
|  |  | 5.People-centredness | | | 5.1. HCP communication / listening skills; 5.4. HCP respect, empathy and friendliness. |  |
|  |  | 6.Safety | | | 6.1. Safety of care and of care spaces (including patient segregation if/when necessary). |  |
|  |  | 9.Overall perceived quality of care | | | 9.1. Technical/Instrumental quality of care: Perceived skills of the care team (including knowledge levels / expertise of the healthcare professionals providing care). |  |
|  |  | 11.Service aspect of care | | | 11.1. Premises/ Facilities (cleanliness, toilets, seating, room design and furnishing, functionality of care spaces, play areas, availability of food/snacks). |  |
| **Q20. Quality improvement program survey in CF care**  Developed and applied by: (49) | Yes; CF | **Total = 7/11** | | | **Total = 11/71** | Partially |
|  |  | 1.Access | | | 1.7. How the appointment was made (in person, by phone, online) – easiness. |  |
|  |  | 2.Comprehensiveness | | | 2.1. Access to multidisciplinary care; 2.8. Access to/existence of clinical care guidelines and necessary/optimal testing/monitoring. |  |
|  |  | 4.Coordination | | | 4.1. Team working of the healthcare professionals involved in care (including case conferences); 4.2. Effectiveness of how care is coordinated; 4.6. Access to medical records/ history/ registries; 4.7. All specialists needed to address the case were present during consultations. |  |
|  |  | 5.People-centredness | | | 5.3. Preferences of patient taken into account / involved in decision-making. |  |
|  |  | 6.Safety | | | 6.1. Safety of care and of care spaces (including patient segregation if/when necessary). |  |
|  |  | 7.Self-management support | | | 7.7. Education/self-management programmes. |  |
|  |  | 9.Overall perceived quality of care | | | 9.1. Technical/Instrumental quality of care: Perceived skills of the care team (including knowledge levels / expertise of the healthcare professionals providing care). |  |
| **Q21. CF Trust Standards of care**  Developed and applied by: (34) | Yes; CF | **Total = 7/11** | | | **Total = 11/71** | No |
|  |  | 1.Access | | | 1.4. Location of appointments – convenience. |  |
|  |  | 2.Comprehensiveness | | | 2.1. Access to multidisciplinary care; 2.4. Peer-support (patient organisation or other): existing peer-support, access to peer-support. |  |
|  |  | 3.Continuity | | | 3.1. Is there a named healthcare professional who they can contact / how contactable are they. |  |
|  |  | 4.Coordination | | | 4.1. Team working of the healthcare professionals involved in care (including case conferences); 4.3. Given a care plan (written / digital); 4.4. Transfer of information to the local doctor: specialist / primary care central / GP (between hospitals/clinics). |  |
|  |  | 5.People-centredness | | | 5.1. HCP communication / listening skills. |  |
|  |  | 6.Safety | | | 6.2. Infection prevention measures, e.g. hand hygiene. |  |
|  |  | 11.Service aspect of care | | | 11.1. Premises/ Facilities (cleanliness, toilets, seating, room design and furnishing, functionality of care spaces, play areas, availability of food/snacks); 11.5. Received reminders for appointments |  |
| **Q22. Expectations and needs of CF patients and the CF center’s response**  Developed and applied by: (38) | Yes; CF | **Total = 6/11** | | | **Total = 8/71** | No |
|  |  | 1.Access | | | 1.5. Mode of appointment (in person, phone, video call) – acceptability; 1.7. How the appointment was made (in person, by phone, online) – easiness. |  |
|  |  | 3.Continuity | | | 3.1. Is there a named healthcare professional who they can contact / how contactable are they; 3.2. Follow-up path planned after the visit/ hospital discharge (including booking of regular examinations...). |  |
|  |  | 4.Coordination | | | 4.3. Given a care plan (written / digital). |  |
|  |  | 5.People-centredness | | | 5.1. HCP communication / listening skills. |  |
|  |  | 7.Self-management support | | | 7.2. Information on treatment (clear info on objectives, results...). |  |
|  |  | 10.Diagnosis & treatment/interventions | | | 10.2. Access to treatment (medication refills, emergency medication, inpatient treatment, home treatment, local hospital, bureaucracy). |  |
| **Q23. CF adherence promotion and satisfaction with care**  Developed and applied by: (32) | Yes; CF | **Total = 5/11** | | | **Total = 7/71** | No |
|  |  | 4.Coordination | | | 4.3. Given a care plan (written / digital). |  |
|  |  | 5.People-centredness | | | 5.4. HCP respect, empathy and friendliness. |  |
|  |  | 7.Self-management support | | | 7.2. Information on treatment (clear info on objectives, results...); 7.8. Information on everyday management and daily life (sex life, sports). |  |
|  |  | 9.Overall perceived quality of care | | | 9.2. Overall satisfaction with care; 9.3. Overall experience with the care received. |  |
|  |  | 10.Diagnosis & treatment/interventions | | | 10.5. Adherence to treatment (e.g. discussing/addressing barriers to adherence) |  |
| **Q24. CF Ward Round Survey**  Developed and applied by: (35) | Yes; CF | **Total = 3/11** | | | **Total = 6/71** | No |
|  |  | 1.Access | | | 1.1. Duration of appointments (little time/enough time); 1.4. Timing of appointments - suitability; 1.4. Location of appointments -convenience. |  |
|  |  | 5.People-centredness | | | 5.1. HCP communication / listening skills; 5.2. Patient/carer participation/involvement in care decision-making; 5.4. HCP respect, empathy and friendliness. |  |
|  |  | 7.Self-management support | | | Other: only mention info provision. Not further specified. |  |
| **Q25. Disease Experiences of Minority Adults With CF**  Developed and applied by: (36) | Yes; (CF) | **Total = 3/11** | | | **Total = 4/71** | Partially |
|  |  | 7.Self-management support | | | 7.1. Information provided on the rare condition (before /during / after diagnosis). |  |
|  |  | 9. Overall perceived quality of care | | | 9.2. Overall satisfaction with care; 9.3. Overall experience with the care received. |  |
|  |  | 10.Diagnosis & treatment/interventions | | | 10.1. Diagnosis (timely, misdiagnosis, length of diagnosis journey, emotional impact, feeling dismissed). |  |
| **Q26. CF related adaptation of the Foundation Trusts Friends and Family Test**  Developed and applied by: (33) | Yes; CF | **Total = 2/11** | | | **Total = 4/71** | Partially |
|  |  | 5.People-centredness | | | 5.1. HCP communication / listening skills; 5.2. Patient/carer participation/involvement in care decision-making; 5.4. HCP respect, empathy and friendliness. |  |
|  |  | 9.Overall perceived quality of care | | | 9.3. Overall experience with the care received. |  |
| **Q27. Partnership between parents of children with CF and the CF team**  Developed and applied by: (45) | Yes; CF | **Total = 1/11** | | | **Total = 3/71** | Yes |
|  |  | 5.People-centredness | | | 5.1. HCP communication / listening skills; 5.2. Patient/carer participation/involvement in care decision-making; 5.3. Preferences of patient taken into account / involved in decision-making. |  |
| **Q28. End-of-Life Care Quality for Children With Cancer**  Developed and applied by: (37) | Yes; Paediatric cancer | **Total = 5/11** | | | **Total = 12/71** | Partially |
|  |  | 1.Access | | | 1.4. Location of appointments – convenience. |  |
|  |  | 2.Comprehensiveness | | | 2.1. Access to multidisciplinary care; 2.2. Psychological/emotional support (patient and/or carer); 2.6. Guidance about social services and education resources; 2.11. Supply/access to medical/physical aid equipment, home care; 2.12. Respite care and bereavement support (for family carers). |  |
|  |  | 4.Coordination | | | 4.3. Given a care plan (written / digital). |  |
|  |  | 5.People-centredness | | | 5.1. HCP communication / listening skills; 5.2. Patient/carer participation/involvement in care decision-making; 5.3. Preferences of patient taken into account / involved in decision-making. |  |
|  |  | 7.Self-management support | | | 7.1. Information provided on the rare condition (before /during / after diagnosis); 7.3. Quality of the information provided (accessibility, effectiveness / accuracy, timeliness, understandability, format). |  |
| **Q29. Survey on high quality, patient centred and coordinated care for Alstrom syndrome**  Developed and applied by: (55) | Yes; Alstrom syndrome | **Total=9/11** | | **Total = 23/71** | | No |
|  |  | 1.Access | | 1.1. Duration of appointments (little time/enough time); 1.4. Timing of appointments – suitability. | |  |
|  |  | 2. Comprehensiveness | | 2.1. Access to multidisciplinary care; 2.2. Psychological/emotional support (patient and/or carer); 2.3. Access to specialized providers; 2.4. Peer-support (patient organisation or other): existing peer-support, access to peer-support. | |  |
|  |  | 3.Continuity | | 3.3. Care continuity. | |  |
|  |  | 4.Coordination | | 4.1. Team working of the healthcare professionals involved in care (including case conferences); 4.3. Given a care plan (written / digital); 4.4. Transfer of information to the local doctor: specialist / primary care central / GP (between hospitals/clinics); 4.7. All specialists needed to address the case were present during consultations. | |  |
|  |  | 5.People-centredness | | 5.1. HCP communication / listening skills; 5.2. Patient/carer participation/involvement in care decision-making; 5.3. Preferences of patient taken into account / involved in decision-making. | |  |
|  |  | 7.Self-management support | | 7.1. Information provided on the rare condition (before /during / after diagnosis); 7.10. Information on research and registries; 7.4. Peer-support (patient organisation or other): informed of availability, given contact; 7.5. Information on where to turn with questions (person / website / support group); 7.7. Education/self-management programmes. | |  |
|  |  | 8.Trust | | 8.1. Trust / confidence in the care team. | |  |
|  |  | 9.Overall perceived quality of care | | 9.1. Technical/Instrumental quality of care: Perceived skills of the care team (including knowledge levels / expertise of the healthcare professionals providing care); 9.4. Patient participation in research | |  |
|  |  | 10.Diagnosis & treatment/interventions | | 10.3. Treatment effectiveness and satisfaction (existence and/or access to guidelines or other validated tools to assess and monitor response)**.** | |  |
| **Q30. Poland syndrome patient experiences with diagnosis and care**  Developed and applied by: (47) | Yes; Poland syndrome | **Total = 9/11** | | | **Total = 14/71** | Yes |
|  |  | 1.Access | | | 1.6. Insurance coverage and financial burden (parking, travelling, testing costs, access other types of financial support and reimbursement of care). |  |
|  |  | 2. Comprehensiveness | | | 2.1. Access to multidisciplinary care; 2.3. Access to specialized provider; 2.4. Peer-support (patient organisation or other): existing peer-support, access to peer-support; 2.6. Guidance about social services and education resources. |  |
|  |  | 3.Continuity | | | 3.1. Is there a named healthcare professional who they can contact / how contactable are they. |  |
|  |  | 4.Coordination | | | 4.1. Team working of the healthcare professionals involved in care (including case conferences). |  |
|  |  | 5.People-centredness | | | 5.1. HCP communication / listening skills. |  |
|  |  | 7.Self-management support | | | 7.4. Peer-support (patient organisation or other): informed of availability, given contact; 7.13. Information on reimbursement of care. |  |
|  |  | 9.Overall perceived quality of care | | | 9.4. Patient participation in research |  |
|  |  | 10.Diagnosis & treatment/interventions | | | 10.1. Diagnosis (timely, misdiagnosis, length of diagnosis journey, emotional impact, feeling dismissed); 10.3. Treatment effectiveness and satisfaction (existence and/or access to guidelines or other validated tools to assess and monitor response) |  |
|  |  | 11.Service aspect of care | | | 11.4. Administrative aspects of care |  |
| **Q31. Systemic sclerosis (SS) patient experience with healthcare and perceived care quality**  Developed and applied by: (52–54) | Yes; SS | **Total = 8/11** | | | **Total = 19/71** |  |
|  |  | 1.Access | | | 1.1. Duration of appointments (little time/enough time); 1.4. Location of appointments – convenience. | Partially |
|  |  | 2. Comprehensiveness | | | 2.1. Access to multidisciplinary care; 2.3. Access to specialised providers; 2.8. Access to/existence of clinical care guidelines and necessary/optimal testing/monitoring. |  |
|  |  | 3.Continuity | | | 3.2. Follow-up path planned after the visit/ hospital discharge (including booking of regular examinations...). |  |
|  |  | 4.Coordination | | | 4.1. Team working of the healthcare professionals involved in care (including case conferences); 4.3. Given a care plan (written / digital); 4.4. Transfer of information to the local doctor: specialist / primary care central / GP (between hospitals/clinics). |  |
|  |  | 5.People-centredness | | | 5.1. HCP communication / listening skills; 5.2. Patient/carer participation/involvement in care decision-making; 5.3. Preferences of patient taken into account / involved in decision-making; 5.4. HCP respect, empathy and friendliness. |  |
|  |  | 7.Self-management support | | | 7.3. Quality of the information provided (accessibility, effectiveness / accuracy, timeliness, understandability, format); 7.4. Peer-support (patient organisation or other): informed of availability, given contact; 7.5. Information on where to turn with questions (person / website / support group). |  |
|  |  | 9.Overall perceived quality of care | | | 9.1. Technical/Instrumental quality of care: Perceived skills of the care team (including knowledge levels / expertise of the healthcare professionals providing care). |  |
|  |  | 10.Diagnosis & treatment/interventions | | | 10.1. Diagnosis (timely, misdiagnosis, length of diagnosis journey, emotional impact, feeling dismissed); 10.3. Treatment effectiveness and satisfaction (existence and/or access to guidelines or other validated tools to assess and monitor response). |  |
| **Q32. Quality of Care of Patients with Chronic Lymphoedema**  Developed and applied by: (51) | No, not exclusively but tested in Primary lymphoedema. | **Total = 7/11** | | | **Total = 13/71** |  |
|  |  | 1. Access | | | 1.1. Duration of appointments (little time/enough time). | Partially |
|  |  | 2. Comprehensiveness | | | 2.3. Access to specialised providers; 2.4. Peer-support (patient organisation or other): existing peer-support, access to peer-support. |  |
|  |  | 4.Coordination | | | 4.1. Team working of the healthcare professionals involved in care (including case conferences). |  |
|  |  | 5.People-centredness | | | 5.2. Patient/carer participation/involvement in care decision-making. |  |
|  |  | 7.Self-management support | | | 7.1. Information provided on the rare condition (before /during / after diagnosis); 7.2. Information on treatment (clear info on objectives, results...); 7.7. Education/self-management programmes; 7.8. Information on everyday management and daily life (sex life, sports). |  |
|  |  | 9.Overall perceived quality of care | | | 9.2. Overall satisfaction with care; 9.3. Overall experience with the care received. |  |
|  |  | 10.Diagnosis & treatment/interventions | | | 10.4. Access to appropriate diagnostic testing (e.g. genetic testing); 10.2. Access to treatment (medication refills, emergency medication, inpatient treatment, home treatment, local hospital, bureaucracy); |  |
| **Q33. Physical, emotional**  **and financial impact of myelofibrosis**  Developed and applied by: (46) | Yes; Myelofibrosis | **Total = 7/11** | | | **Total = 11/71** | Partially |
|  |  | 1.Access | | | 1.1. Duration of appointments (little time/enough time); 1.4. Location of appointments - convenience; 1.6. Insurance coverage and financial burden (parking, travelling, testing costs, access other types of financial support and reimbursement of care). |  |
|  |  | 5.People-centredness | | | 5.1. HCP communication / listening skills; 5.4. HCP respect, empathy and friendliness. |  |
|  |  | 7.Self-management support | | | 7.1. Information provided on the rare condition (before /during / after diagnosis). |  |
|  |  | 8.Trust | | | 8.1. Trust / confidence in the care team. |  |
|  |  | 9.Overall perceived quality of care | | | 9.1. Technical/Instrumental quality of care: Perceived skills of the care team (including knowledge levels / expertise of the healthcare professionals providing care). |  |
|  |  | 10.Diagnosis & treatment/interventions | | | 10.1. Diagnosis (timely, misdiagnosis, length of diagnosis journey, emotional impact, feeling dismissed). 10.7. Discomfort in pre-diagnosis phase of care; |  |
|  |  | 11.Service aspect of care | | | 11.1. Premises/ Facilities (cleanliness, toilets, seating, room design and furnishing, functionality of care spaces, play areas, availability of food/snacks. |  |
| **Q34. Maternal Reports of Satisfaction With Care and Outcomes for Children With Craniosynostosis**  Developed and applied by: (40) | Yes; Craniosynostosis | **Total = 7/11** | | | **Total = 11/71** | Partially |
|  |  | 1.Access | | | 1.6. Insurance coverage and financial burden (parking, travelling, testing costs, access other types of financial support and reimbursement of care). |  |
|  |  | 2. Comprehensiveness | | | 2.1. Access to multidisciplinary care; 2.2. Psychological/emotional support (patient and/or carer); 2.3. Access to specialized providers; 2.9. Liaison and collaboration with schools and school-related services (awareness, liaison with school nurses, learning interventions, systematic instructions in school by HCPs). |  |
|  |  | 3.Continuity | | | 3.1. Is there a named healthcare professional who they can contact / how contactable are they. |  |
|  |  | 4.Coordination | | | 4.1. Team working of the healthcare professionals involved in care (including case conferences). |  |
|  |  | 5.People-centredness | | | 5.1. HCP communication / listening skills. |  |
|  |  | 7.Self-management support | | | 7.1. Information provided on the rare condition (before /during / after diagnosis); 7.2. Information on treatment (clear info on objectives, results...). |  |
|  |  | 10.Diagnosis & treatment/interventions | | | 10.3. Treatment effectiveness and satisfaction (existence and/or access to guidelines or other validated tools to assess and monitor response); 10.6. Treatment safety (e.g long-time/severe side effects and/or adverse reactions). |  |
| **Q35. Haemophilia Treatment Center (HTC) - National Patient Satisfaction Survey**  Developed and applied by: (39) | Yes; Haemophilia and Von Willebrand disease | **Total = 6/11** | | | **Total = 13/71** | Yes |
|  |  | 1.Access | | | 1.1. Duration of appointments (little time/enough time); 1.2. Waiting time (in the waiting room, to get an appointment after doing tests, outside of routine visits, unexpected cancellations); 1.6. Insurance coverage and financial burden (parking, travelling, testing costs, access other types of financial support and reimbursement of care). |  |
|  |  | 2.Comprehensiveness | | | 2.1. Access to multidisciplinary care; 2.2. Psychological/emotional support (patient and/or carer); 2.5. Involvement of/access to social worker. |  |
|  |  | 4.Coordination | | | 4.2. Effectiveness of how care is coordinated; 4.4. Transfer of information to the local doctor: specialist / primary care central / GP (between hospitals/clinics). |  |
|  |  | 5.People-centredness | | | 5.1. HCP communication / listening skills; 5.2. Patient/carer participation/involvement in care decision-making; 5.4. HCP respect, empathy and friendliness. |  |
|  |  | 7.Self-management support | | | 7.3. Quality of the information provided (accessibility, effectiveness / accuracy, timeliness, understandability, format). |  |
|  |  | 9.Overall perceived quality of care | | | 9.2. Overall satisfaction with care. |  |
| **Q36. Mountain States Regional Haemophilia and Thrombosis Centre Patient Satisfaction Survey**  Developed and applied by: (41) | Yes; Haemophilia A and B  Von Willebrand Disease | **Total = 6/11** | | | **Total = 13/71** | Yes |
|  |  | 1.Access | | | 1.1. Duration of appointments (little time/enough time); 1.3. Timing of appointments – suitability; 1.5. Mode of appointment (in person, phone, video call) – acceptability; 1.6. Insurance coverage and financial burden (parking, travelling, testing costs, access other types of financial support and reimbursement of care). |  |
|  |  | 2.Comprehensiveness | | | 2.1. Access to multidisciplinary care; 2.2. Psychological/emotional support (patient and/or carer); 2.4. Peer-support (patient organisation or other): existing peer-support, access to peer-support; 2.6. Guidance about social services and education resources. |  |
|  |  | 5.People-centredness | | | 5.4. HCP respect, empathy and friendliness. |  |
|  |  | 7.Self-management support | | | 7.3. Quality of the information provided (accessibility, effectiveness / accuracy, timeliness, understandability, format). |  |
|  |  | 9.Overall perceived quality of care | | | 9.1. Technical/Instrumental quality of care: Perceived skills of the care team (including knowledge levels / expertise of the healthcare professionals providing care); 9.2. Overall satisfaction with care. |  |
|  |  | 11.Service aspect of care | | | 11.1. Premises/ Facilities (cleanliness, toilets, seating, room design and furnishing, functionality of care spaces, play areas, availability of food/snacks). |  |
| **Q37. Quality of care for multiple endocrine neoplasia disorders**  Developed and applied by: (50) | Yes; Multiple endocrine neoplasia (MEN) | **Total = 6/11** | | | **Total = 7/71** | Partially |
|  |  | 1.Access | | | 1.1. Duration of appointments (little time/enough time). |  |
|  |  | 2. Comprehensiveness | | | 2.3. Access to specialised providers. |  |
|  |  | 5.People-centredness | | | 5.1. HCP communication / listening skills; 5.2. Patient/carer participation/involvement in care decision-making. |  |
|  |  | 8.Trust | | | 8.1. Trust / confidence in the care team. |  |
|  |  | 9.Overall perceived quality of care | | | 9.1. Technical/Instrumental quality of care: Perceived skills of the care team (including knowledge levels / expertise of the healthcare professionals providing care); 9.2. Overall satisfaction with care. |  |
|  |  | 10.Diagnosis & treatment/interventions | | | 10.4. Access to appropriate diagnostic testing (e.g. genetic testing). |  |
| **Q38. Parental experience of, and satisfaction with, our tertiary paediatric service for children with Non-cystic fibrosis bronchiectasis (NCFB) and (primary ciliary dyskinesia (PCD)**  Developed and applied by: (44) | Yes; NCFB, PCD | **Total = 5/11** | | | **Total = 8/71** | No |
|  |  | 1.Comprehensiveness | | | 2.1. Access to multidisciplinary care. |  |
|  |  | 3.Continuity | | | 3.1. Is there a named healthcare professional who they can contact / how contactable are they; 3.4. Access to and satisfaction with emergency care; 3.6. Have an alert card about their condition which can be used in emergency situations / when they are in contact with a new healthcare professional. |  |
|  |  | 5.People-centredness | | | 5.1. HCP communication / listening skills. |  |
|  |  | 7.Self-management support | | | 7.5. Information on where to turn with questions (person / website / support group); 7.9. Information on elective admissions and upon hospital discharge. |  |
|  |  | 9.Overall perceived quality of care | | | 9.1. Technical/Instrumental quality of care: Perceived skills of the care team (including knowledge levels / expertise of the healthcare professionals providing care) |  |
| **Q39. Primary Biliary Cholangitis (PBC) patient reported experiences * app-based PREMs**  Developed and applied by: (48) | Yes; PBC | **Total = 4/11** | | | **Total = 6/71** | Partially |
|  |  | 2.Comprehensiveness | | | 2.1. Access to multidisciplinary care. |  |
|  |  | 5.People-centredness | | | 5.2. Patient/carer participation/involvement in care decision-making; 5.3. Preferences of patient taken into account / involved in decision-making. |  |
|  |  | 7.Self-management support | | | 7.1. Information provided on the rare condition (before /during / after diagnosis); 7.2. Information on treatment (clear info on objectives, results...). |  |
|  |  | 10.Diagnosis & treatment/interventions | | | 10.3. Treatment effectiveness and satisfaction (existence and/or access to guidelines or other validated tools to assess and monitor response). |  |
| **Q40. Paediatric hereditary angioedema patient experience survey**  Developed and applied by: (42) | Yes; Paediatric hereditary angioedema | **Total = 3/11** | | | **Total = 5/71** | Yes |
|  |  | 2.Comprehensiveness | | | 2.3. Access specialized providers; 2.9. Liaison and collaboration with schools and school-related services (awareness, liaison with school nurses, learning interventions, systematic instructions in school by HCPs). |  |
|  |  | 3.Continuity | | | 3.6. Have an alert card about their condition which can be used in emergency situations / when they are in contact with a new healthcare professional. |  |
|  |  | 10.Diagnosis & treatment/interventions | | | 10.1. Diagnosis (timely, misdiagnosis, length of diagnosis journey, emotional impact, feeling dismissed); 10.2. Access to treatment (medication refills, emergency medication, inpatient treatment, home treatment, local hospital, bureaucracy). |  |
| **Q41. Patient experience with healthcare transition from childhood to adulthood in pseudoxanthoma elasticum**  Developed and applied by: (43) | Yes; Pseudoxanthoma elasticum | **Total = 3/11** | | | **Total = 4/71** | Partially |
|  |  | 2.Comprehensiveness | | | 2.1. Access to multidisciplinary care. |  |
|  |  | 3.Continuity | | | 3.2. Follow-up path planned after the visit/ hospital discharge (including booking of regular examinations…); 3.5. Access to and satisfaction with transition of paediatric to adult care. |  |
|  |  | 4.Coordination | | | 4.1. Team working of the healthcare professionals involved in care (including case conferences). |  |
| **Q42. Alkaptonuria patient experiences of receiving diagnosis and care**  Developed and applied by: (31) | Yes ; Alkaptonuria | **Total = 3/11** | | | **Total = 3/71** | Yes |
|  |  | 7.Self-f-management support | | | 7.1. Information provided on the rare condition (before /during / after diagnosis). |  |
|  |  | 9.Overall perceived quality of care | | | 9.1. Technical/Instrumental quality of care: Perceived skills of the care team (including knowledge levels / expertise of the healthcare professionals providing care). |  |
|  |  | 10.Diagnosis & treatment/interventions | | | 10.1. Diagnosis (timely, misdiagnosis, length of diagnosis journey, emotional impact, feeling dismissed). |  |
|  |  |  | | |  |  |

**Legend:** ACE - Adult Cystic fibrosis Experience; CF – Cystic fibrosis; CHC-SUN - Child Health Care Questionnaire – Satisfaction Utilization and Needs; CSQ-4 - Customer satisfaction questionnaire; GCSS - Genetic Counselling Satisfaction Scale; GP – General practitioner; HCP – Healthcare professional; HTC - Haemophilia Treatment Center; IBD - Inflammatory bowel disease; IEXPAC - Chronic Patient Experience Evaluation Instrument; NCFB - Non-cystic fibrosis bronchiectasis; PAHC-PREM - Patient’s Experiences of Health Care in Pulmonary Arterial Hypertension Outpatient Clinics; PBC - Primary Biliary Cholangitis; PCD - Primary Ciliary Dyskinesia; SCNS-SF34 - Supportive Care Needs Survey Short Form; SS – Systemic sclerosis; YHC-SUN - Youth Healthcare measure-satisfaction, utilization, and needs

**References**

1.	Schmidt S, Thyen U, Chaplin J, Mueller-Godeffroy E. Cross-cultural development of a child health care questionnaire on satisfaction, utilization, and needs. Ambul Pediatr. 2007;7(5):374–82.

2.	Schmidt S, Thyen U, Chaplin J, Mueller-Godeffroy E, Bullinger M. Healthcare needs and healthcare satisfaction from the perspective of parents of children with chronic conditions: the DISABKIDS approach towards instrument development. Child Care Health Dev. 2008;34(3):355–66.

3.	Chrysochoou EA, Hatziagorou E, Tsanakas J. Healthcare satisfaction, utilisation, and needs among families with cystic fibrosis. Journal of Cystic Fibrosis. 2019;18(Supplement 1):S174-.

4.	Schmidt S, Thyen U, Herrmann-Garitz C, Bomba F, Muehlan H. The Youth Health Care measure-satisfaction, utilization, and needs (YHC-SUN)-development of a self-report version of the Child Health Care (CHC-SUN) proxy-measure. BMC Health Serv Res. 2016;16:189.

5.	Attkisson CC, Larsen D. 1978. [cited 2024 Feb 13]. Client Satisfaction Questionnaire (CSQ). Available from: https://eprovide.mapi-trust.org/instruments/client-satisfaction-questionnaire

6.	Thyen U, Ittermann T, Flessa S, Muehlan H, Birnbaum W, Rapp M, et al. Quality of health care in adolescents and adults with disorders/differences of sex development (DSD) in six European countries (dsd-LIFE). BMC Health Serv Res. 2018;18(1):527.

7.	Gidengil C, Parast L, Burkhart ; Q, Brown J, Elliott MN, Lion ; K Casey, et al. Development and Implementation of the Family Experiences With Coordination of Care Survey Quality Measures [Internet]. Vol. 17, ACADEMIC PEDIATRICS. 2017. Available from: http://www.seattlechildrens.org/research/child-

8.	deJong NA, Wofford M, Song PH, Kappelman MD. Association of Care Coordination Experience and Health Services Use with Main Provider Type for Children with Inflammatory Bowel Disease. J Pediatr. 2021;234:142-148.e1.

9.	Tercyak KP, Johnson SB, Roberts SF, Cruz AC. Psychological response to prenatal genetic counseling and amniocentesis. Patient Education and Conseling. 2001;43:73–84.

10.	Nevin MS, Barlow-Stewart K, Wakefield EC, McGill CB, LeMarne F, Beavis E, et al. Uncovering the psychosocial impact of undergoing genetic testing for rare and recently discovered genetic epilepsies. Twin Research and Human Genetics. 2021;24(1):71.

11.	Britto MT, Slap GB, DeVellis RF, Hornung RW, Atherton HD, Knopf JM, et al. Specialists understanding of the health care preferences of chronically ill adolescents. J Adolesc Health. 2007;40(4):334–41.

12.	Ellemunter H, Stahl K, Smrekar U, Steinkamp G. Evaluating patient experience in a cystic fibrosis centre using a disease-specific patient satisfaction questionnaire. Eur J Pediatr. 2015;174(11):1451–60.

13.	Boyes A, Girgis A, Lecathelinais C. Brief assessment of adult cancer patients’ perceived needs: development and validation of the 34-item Supportive Care Needs Survey (SCNS-SF34). J Eval Clin Pract [Internet]. 2009 Aug 1 [cited 2024 Jan 22];15(4):602–6. Available from: https://onlinelibrary.wiley.com/doi/full/10.1111/j.1365-2753.2008.01057.x

14.	Husson O, Weidema M, Leonard H, Hartle DeYoung L, van der Graaf W, van de Poll‐Franse L. Supportive care needs of patients living with an extremely rare and unpredictable cancer: The Epithelioid Haemangioendothelioma patient experience. Eur J Cancer Care (Engl) [Internet]. 2021;30(6):1–11. Available from: https://search.ebscohost.com/login.aspx?direct=true&db=cin20&AN=153493799&site=ehost-live

15.	Absolom K, Greenfield D, Ross R, Horne B, Davies H, Glaser A, et al. Predictors of clinic satisfaction among adult survivors of childhood cancer. Eur J Cancer. 2006 Jul;42(10):1421–7.

16.	van Laar M, Glaser A, Phillips RS, Feltbower RG, Stark DP. The impact of a managed transition of care upon psychosocial characteristics and patient satisfaction in a cohort of adult survivors of childhood cancer. Psychooncology. 2013;22(9):2039–45.

17.	Steinkamp G, Stahl K, Ullrich G, Heuer E, Ellemunter H, Schwarz C, et al. CF care through the patient’s eyes - Development of a disease specific questionnaire measuring patient satisfaction with CF services. Journal of Cystic Fibrosis. 2010;9(SUPPL. 1):S100-.

18.	Steinkamp G, Stahl K, Ellemunter H, Heuer E, S van KR, Busche M, et al. Cystic fibrosis (CF) care through the patients’ eyes - a nationwide survey on experience and satisfaction with services using a disease-specific questionnaire. Respir Med. 2015;109(1):79–87.

19.	Stahl K, Steinkamp G, Ullrich G, Schulz W, S van KR, Heuer HE, et al. Patient experience in cystic fibrosis care: Development of a disease-specific questionnaire. Chronic Illn. 2015;11(2):108–25.

20.	Homa K, Sabadosa KA, Nelson EC, Rogers WH, Marshall BC. Development and validation of a cystic fibrosis patient and family member experience of care survey. Qual Manag Health Care [Internet]. 2013 Apr [cited 2024 Jan 22];22(2):100–16. Available from: https://journals.lww.com/qmhcjournal/fulltext/2013/04000/development_and_validation_of_a_cystic_fibrosis.3.aspx

21.	Homa K, Sabadosa KA, Marrow LC, Marshall BC. Experience of care from the perspective of individuals with cystic fibrosis and families: Results from 70 CF Foundation accredited programs in the USA. J Cyst Fibros. 2015;14(4):515–22.

22.	Waldréus N, Jaarsma T, Ivarsson B, Strömberg A, Årestedt K, Kjellström B. Development and Validation of a Questionnaire to Measure Patient’s Experiences of Health Care in Pulmonary Arterial Hypertension Outpatient Clinics. Heart Lung Circ. 2019;28(7):1074–81.

23.	Sadlo A, Altevers J, Peplies J, Kaltz B, Claßen M, Bauer A, et al. Measuring satisfaction with health care in young persons with inflammatory bowel disease--an instrument development and validation study. BMC Health Serv Res. 2014;14:97.

24.	Al-Yateem N, Docherty C, Rossiter R. Determinants of Quality of Care for Adolescents and Young Adults With Chronic Illnesses: A Mixed Methods Study. J Pediatr Nurs. 2016;31(3):255–66.

25.	Hytiris M, Johnston D, Mullen S, Smyth A, Dougan E, Rodie M, et al. Experience of health care at a reference centre as reported by patients and parents of children with rare conditions. Orphanet J Rare Dis. 2021;16(1):65.

26.	Guilabert M, Martínez-García A, Sala-González M, Solas O, Mira JJ. Results of a Patient Reported Experience Measure (PREM) to measure the rare disease patients and caregivers experience: a Spanish cross-sectional study. Orphanet J Rare Dis. 2021;16(1):67.

27.	Jarvis C, Thompson A, Marshall L, Fletcher N, Minson S. Patient experiences of transition from paediatric to adult services. Arch Dis Child. 2021;106(SUPPL 1):A240-.

28.	Spencer-Tansley R, Meade N, Ali F, Simpson A, Hunter A. Mental health care for rare disease in the UK - recommendations from a quantitative survey and multi-stakeholder workshop. BMC Health Serv Res. 2022;22(1):648.

29.	D’Aprano A, Gibb S, Riess S, Cooper M, Mountford N, Meehan E. Important components of a programme for children with medical complexity: An Australian perspective. Child Care Health Dev. 2020;46(1):90–103.

30.	Finlayson F. A New, Short Patient-Reported Experience Measure (Prem) for Cystic Fibrosis (CF) Care a- the Adult Cystic Fibrosis Experience (ACE) Measure. Respirology. 2020;25((Finlayson) Respiratory Medicine, Alfred, Melbourne, VIC, Australia):154.

31.	Rudebeck M, Scott C, Sireau N, Ranganath L. A patient survey on the impact of alkaptonuria symptoms as perceived by the patients and their experiences of receiving diagnosis and care. JIMD Rep. 2020 May 1;53(1):71–9.

32.	Riekert KA, Eakin MN, Bilderback A, Ridge AK, Zhang J, Quittner AL. Adolescent and parent perceptions of their CF care team’s adherence promotion practices and satisfaction with care. Pediatr Pulmonol. 2012;47(SUPPL. 35):393–4.

33.	Thomas A, Bradley J, Pickering N, Saba T. Patient experience of transition to a newly established cystic fibrosis service in the North West of England. Journal of Cystic Fibrosis. 2019;18(Supplement 1):S188-.

34.	Berry L, Newson TP, Smith O. Benefits of patient feedback in quality improvement of a local cystic fibrosis service. Arch Dis Child. 2019;104(Supplement 2):A207–8.

35.	MacDonald K, Mathews A. Exploring experiences of the in-patient ward round with young people with CF. Journal of Cystic Fibrosis. 2017;16(Supplement 1):S163-.

36.	Hutchins K, Barr E, Bellcross C, Ali N, Guan Y, Hunt W. Evaluating potential differences in the disease experiences of adult minority patients with cystic. fibrosis. Journal of Cystic Fibrosis. 2021;20(Supplement 2):S141-.

37.	Ananth P, Mun S, Reffat N, Kang SJ, Pitafi S, Ma X, et al. Refining Patient-Centered Measures of End-of-Life Care Quality for Children With Cancer. JCO Oncol Pract. 2022;18(3):e372–82.

38.	Cafaggi M, Marcucci M, Festini F, Braggion C. Expectations and needs of cystic fibrosis (CF) patients and the CF center’s response. Journal of Cystic Fibrosis. 2011;10(SUPPL. 1):S93-.

39.	Riske B, Shearer R, Baker JR. Patient satisfaction with US Hemophilia Treatment Center Care, Teams and Services: The First National Survey. Haemophilia. 2020 Nov 1;26(6):991–8.

40.	Wong-Gibbons DL, Kancherla V, Romitti PA, Tyler MC, Damiano PC, Druschel CM, et al. Maternal reports of satisfaction with care and outcomes for children with craniosynostosis. J Craniofac Surg. 2009;20(1):138–42.

41.	Hacker MR, Primeaux J, Manco-Johnson MJ. A patient satisfaction survey for haemophilia treatment centres. Haemophilia. 2006;12(2):163–8.

42.	Read N, Lim E, Tarzi MD, Hildick-Smith P, Burns S, Fidler KJ. Paediatric hereditary angioedema: a survey of UK service provision and patient experience. Clin Exp Immunol. 2014;178(3):483–8.

43.	Lechevalier D, Sigg N, Humeau H, Vermersch C, Leducq S, Maruani A, et al. Healthcare transition from childhood to adulthood in pseudoxanthoma elasticum: Patient experience and recommendations for health practitioners. Ann Dermatol Venereol. 2022;

44.	Sharif N, Shawcross A, Tahsin E, Davey S, Maitra A. Insights into parental experience of a specialist bronchiectasis and primary ciliary dyskinesia service provision-experience from a large tertiary paediatric centre in United Kingdom. Thorax. 2021;76(SUPPL 1):A157-.

45.	Heeres I. Partnership between parents of children with CF and the CF team at the Erasmus MC-sophia children’s hospital. Pediatr Pulmonol. 2015;50(SUPPL. 41):439.

46.	Palandri F, Benevolo G, Iurlo A, Abruzzese E, Carella AM, Paoli C, et al. Life for patients with myelofibrosis: the physical, emotional and financial impact, collected using narrative medicine-Results from the Italian “Back to Life” project. Qual Life Res. 2018;27(6):1545–54.

47.	Baldelli I, Gallo F, Crimi M, Fregatti P, Mellini L, Santi P, et al. Experiences of patients with Poland syndrome of diagnosis and care in Italy: a pilot survey. Orphanet J Rare Dis. 2019;14(1):269.

48.	Mitchell-Thain R, Yeoman A, Hegade V. Patient reported experiences through PBC foundation app: What impacts upon a PBC patient’s. experience? Gut. 2021;70(SUPPL 1):A39-.

49.	Pougheon Bertrand D, Minguet G, Gagnayre R, Lombrail P. Lessons from patient and parent involvement (P&PI) in a quality improvement program in cystic fibrosis care in France. Orphanet J Rare Dis. 2018;13(Suppl 1):19.

50.	Drewitz KP, Grey J, Brügmann P, Pichl J, Sammarco M, Aarts M, et al. Patients’ perception on the quality of care for multiple endocrine neoplasia disorders in Europe: an online survey from a patient support group. Endocrine. 2021;71(3):634–40.

51.	Herberger K, Blome C, Sandner A, Altheide F, Heyer K, Münter KC, et al. Quality of care of patients with chronic lymphoedema in Germany. Dermatology. 2013;226(3):238–46.

52.	Spierings J, C van den E, Schriemer R, L de P, Moens HB, J van L, et al. Optimal care for systemic sclerosis patients: recommendations from a patient-centered and multidisciplinary mixed-method study and working conference. Clin Rheumatol. 2019;38(4):1007–15.

53.	Spierings J, H VDEC, Schriemer MR, K DVBJ, Vonk MC. Patients’ evaluation of Dutch health care in systemic sclerosis: Unmet needs and preferences. Ann Rheum Dis. 2018;77(Supplement 2):521.

54.	Spierings J, M van den ECH, Schriemer RM, J BMH, A van der BE, Bonte-Mineur F, et al. How do patients with systemic sclerosis experience currently provided healthcare and how should we measure its quality? Rheumatology (Oxford). 2020;59(6):1226–32.

55.	Van Groenendael S, Giacovazzi L, Davison F, Holtkemper O, Huang Z, Wang Q, et al. High quality, patient centred and coordinated care for Alstrom syndrome: a model of care for an ultra-rare disease. Orphanet J Rare Dis. 2015;10:149.
